# Impacts of pro‐inflammatory cytokines variant on cardiometabolic profile and premature coronary artery disease: A systematic review and meta‐analysis

**DOI:** 10.1111/jcmm.18311

**Published:** 2024-04-17

**Authors:** Yang Liu, Yuan Chen, Yi Lin, Baozhu Wei, Zhi Luo

**Affiliations:** ^1^ Department of Endocrinology China Resources and WISCO General Hospital Wuhan China; ^2^ Department of Cardiology, Union Hospital, Tongji Medical College Huazhong University of Science and Technology Wuhan China; ^3^ Department of Cardiology Zhongnan Hospital of Wuhan University Wuhan University Wuhan China; ^4^ Institute of Myocardial Injury and Repair Wuhan University Wuhan China; ^5^ Department of Cardiology Suining Central Hospital, Suining Sichuan China

**Keywords:** Cardiometabolic risk factors, chemokines, coronary artery disease, genetic predisposition to disease

## Abstract

Interleukin‐6 (IL‐6), a pivotal pro‐inflammatory cytokine, is closely linked to vascular wall thickening and atherosclerotic lesion. Since serum IL‐6 levels are largely determined by the genetic variant in *IL‐6*, this study was conducted to investigate whether the *IL‐6* variant impacts cardiometabolic profile and the risk of premature coronary artery disease (PCAD). PubMed, Cochrane Library, Central, Cumulative Index to Nursing and Allied Health Literature (CINAHL), and ClinicalTrials.gov were searched from May 13, 2022 to June 28, 2023. In total, 40 studies (26,543 individuals) were included for the analysis. The rs1800795 (a function variant in the *IL‐6* gene) C allele was linked to higher levels of low‐density lipoprotein cholesterol (LDL‐C), total cholesterol (TC), fasting plasma glucose (FPG), body mass index (BMI), and waist circumference (WC), and a lower levels of high‐density lipoprotein cholesterol (HDL‐C). However, no significant association was observed of rs1800795 with triglycerides (TG), systolic blood pressure (SBP), and diastolic blood pressure (DBP). Interestingly, a significant association was detected between rs1800795 and PCAD. Subgroup analyses indicted that the impacts of rs1800795 on cardiometabolic risk factors were significant in Caucasians but stronger in obese patients. In contrast, the impact of rs1800795 on PCAD was significant in brown race population. In summary, rs1800795 had a slight but significant impact on cardiometabolic risk factors and PCAD. IL‐6 inhibition with ziltivekimab or canakinumab may benefit high‐risk populations (e.g. brown race population, Caucasians, obese patients, etc.) with rs1800795 to prevent PCAD.

## 1 INTRODUCTION

Cytokines‐mediated chronic low‐grade inflammation plays a critical role in coronary artery disease (CAD) pathogenesis.^1^, ^2^ In which, interleukin‐6 (IL‐6) is found to increase the risk of future CAD, nonfatal myocardial infarction (MI), and CAD death^3^, ^4^, ^5^ with a magnitude of effect comparable, if not larger, than that of low‐density lipoprotein cholesterol (LDL‐C).^6^

Serum IL‐6 levels are linked to atherosclerosis. For instance, chronic exposure to high levels of IL‐6 predisposes vascular wall thickening and atherosclerosis.^7^ IL‐6 inhibition with ziltivekimab (an IL‐6 ligand inhibition)^8^, ^9^ and canakinumab [an interleukin‐1β (IL‐1β) ligand inhibition]^10^ disrupted multiple atherogenic inflammatory pathways and inhibited atherosclerosis. In mouse model experiments, IL‐6 mRNA and protein were found to express in atherosclerotic plaques of mice aortas. IL‐6 inhibition with MR16‐1 (a murine anti‐IL‐6 receptor antibody)^11^ and 17beta‐estradiol (a vasculoprotective sex steroid hormone)^12^ reduced atherosclerotic lesions and IL‐6 secretion from ex vivo aortic tissue segments.

The IL‐6 gene is located in the short arm of human chromosome 7 (7p21‐24), including five exons. rs1800795 is located in the promoter at position −174, formed by a transversion from guanine (G) to cytosine (C). The G and C alleles encode low and high activity of IL‐6,^13^ respectively. In addition, the carriers of the C allele had 5‐fold higher serum IL‐6 levels than carriers of the G allele.^14^

Premature coronary artery disease (PCAD) is defined as the first onset of CAD in males less than 55 and females less than 65 years of age.^15^ According to the newest management strategies for PCAD,^16^ smoking, hypertension, diabetes with diabetes‐specific risk‐enhancing factors, severe dyslipidemia, and multiple other risk‐enhancing factors (e.g. obesity, high body mass index [BMI], and large waist circumference [WC]) were recognized as the primary risk factors for PCAD. Since serum IL‐6 levels were primarily determined by rs1800795^17^, ^18^ and were closely linked to CAD^3^, ^4^, ^5^ and atherosclerosis,^7^, ^8^, ^9^, ^10^, ^11^, ^12^ it is tempting to speculate that rs1800795 may influence the risk of CAD and atherosclerosis by modulating serum IL‐6 levels.^3^, ^4^, ^5^, ^7^, ^8^, ^9^, ^10^, ^11^, ^12^, ^17^, ^18^ Since high IL‐6 levels were linked with an increased risk of PCAD,^19^, ^20^ it is tempting to hypothesize that rs1800795 may increase the risk of PCAD by elevating IL‐6 levels. Interestingly, this hypothesis was verified in Ansari et al.^21^ study whereby the C allele of rs1800795 increased the risk of PCAD by enhancing IL‐6 levels. Nevertheless, Sekuri et al.^22^ did not detect a significant association between rs1800795 C allele and PCAD in a Turkish cohort. In contrast, Phulukdaree et al.^23^ claimed that the G allele (but not the C allele) of rs1800795 increased the risk of PCAD in South African Indian men. Since the present results were controversial and inconclusive, this study is required to investigate whether rs1800795 is linked to the risk of PCAD in the light of evidence‐based medicine. If it does, so which allele (C or G) of rs1800795 increases PCAD risk and its underlying mechanisms (i.e. cardiometabolic parameters).

The fulfilment of this study may benefit to prevention and control of PCAD in both healthy individuals and patients with cardiometabolic disorder. For instance, for healthy individuals with a risk allele (C or G) of PCAD, disease prevention could be achieved via lifestyle interventions (e.g. exercise and diet etc.) to weaken potential cardiometabolic risk factors (e.g. lipid, fasting plasma glucose [FPG], blood pressure, BMI, or WC). In contrast, for those with cardiometabolic disorder or PCAD, specific medicine (e.g. lipid‐lowering drugs, hypoglycemic drugs, or antihypertensive drugs) in combination with lifestyle interventions (e.g. exercise and diet etc.) could be initiated to prevent PCAD or multi‐vessel lesions.

Over the last several decades, intensive efforts have been made in the scientific community to search or identify some effective therapeutic targets for PCAD.^16^, ^24^ However, the results were not so satisfactory.^25^ Here, we systematically investigated the impacts of rs1800795 on cardiometabolic risk factors and PCAD in 26,543 individuals, to provide some clues or references for the identification of possible therapeutic target or strategy for PCAD.

## 2 MATERIALS AND METHODS

The current systematic review and meta‐analysis follows the Preferred Reporting Items for Systematic Reviews and Meta‐analyses (PRISMA) 2020 Checklist (http://www.prisma‐statement.org/PRISMAStatement/).^26^ The registration information including registration number is not available (see Table S1 for more details). Studies that meet the following PICOS principle (see Table S2 for more details) are considered preliminary qualified: (I) P (population): healthy individuals or patients in specific ethnicities (e.g. Caucasian, Asian, African, and American etc.); (II) I (intervention): no special interventions, especially currently or in the past 1 month with a medication history of lipid‐lowering drugs, hypoglycemic drugs, or antihypertensive drugs; (III) C (comparison): the studies compare the cardiometabolic characteristics [ie, triglycerides (TG), total cholesterol (TC), LDL‐C, high‐density lipoprotein cholesterol (HDL‐C), FPG, systolic blood pressure (SBP), diastolic blood pressure (DBP), BMI, WC] between carriers of rs1800795 C allele and carriers of rs1800795 G allele; (IV) O (outcome): cardiometabolic parameters are expressed as mean with standard deviation (SD) or standard errors (SE), or the number of genotype in PCAD group and control group is provided, to facilitate the subsequent calculation of standardized mean difference (SMD) and 95% confidence intervals (CI), or risk ratios (RR) and corresponding 95% CI; (V) S (study design): observational study, published in English, and funded by a funding body or institution.

### 2.1 Literature search

A comprehensive literature search was performed from May 13, 2022 to June 28, 2023 using PubMed, Cochrane Library, Central, Cumulative Index to Nursing and Allied Health Literature (CINAHL), and ClinicalTrials.gov. The following main keywords were used in the search: [“IL‐6”] AND [“rs1800795”] OR [“polymorphism”] AND [“lipids,” “blood glucose,” “blood pressure,” “body mass index,” “waist circumference”] AND/OR [“premature coronary artery disease”]. Please see Table S3 for full search strategy and syntaxes.

### 2.2 Inclusion criteria

In addition to meet the PICOS principle, the specific inclusion criteria for the impact of rs1800795 on PCAD include: (I) case–control design; (II) CAD cases were angiographically defined; (III) providing the count of individual genotypes in cases and controls for rs1800795. The inclusion criteria for the impact of rs1800795 on cardiometabolic profile include: (I) studies investigated the association of rs1800795 with lipid, FPG, blood pressure, BMI or WC; (II) studies provided the count of individual genotypes for rs1800795; (III) studies provided mean lipid, FPG, blood pressure, BMI, and WC with SD or SE by the genotype of rs1800795; (IV) interventional studies provided pre‐intervention data; (V) the language of eligible studies was restricted to English. Studies were excluded if any of the following conditions were met. There was no data on genotype distribution in the control group, and the genotype distribution of the control group deviated from the Hardy–Weinberg equilibrium (HWE).

### 2.3 Data extraction

The data extraction were conducted by four investigators (Yang Liu, Yuan Chen, Yi Lin and Baozhu Wei) and cross‐checked by (Zhi Luo). From each included study, the following was extracted: the last name of the first author; publication time; country, race, sex, health status, age, case and control counts, genotype count, study design, study period and mean lipid, blood pressure, FPG, BMI and WC with SD or SE by the genotype of rs1800795.

### 2.4 Data analysis

TG, TC, LDL‐C, HDL‐C and FPG units were converted into mmol/L. Blood pressure units were converted into mmHg. BMI unit was converted into kg/m^2^. WC unit was converted into cm. All extracted data were expressed as mean ± SD. RR and corresponding 95% CI were used to evaluate the strength of rs1800795 in PCAD. SMD with 95% CI was used to evaluate the differences in lipid, blood pressure, FPG, BMI, and WC between the genotype of rs1800795. The pooled RR was performed for the allelic model (*C* vs. *G*), additive model (*CC* vs. *GG*), heterozygote model (*GC* vs. *GG*), dominant model (*GC* + *CC* vs. *GG*), recessive model (*CC* vs. *GG* + *GC*), and overdominant model (*GC* vs. *GG + CC*). Since most of the included studies presented lipid, blood pressure, FPG, BMI, and WC data in a dominant model (*GC* + *CC* vs. *GG*), a dominant model was used to ensure adequate statistical power. All statistical tests were conducted with the Cochrane Collaboration meta‐analysis software (Review Manager 5.4). *p* < 0.05 was recognized as statistically significant.

### 2.5 Subgroup analysis

Subgroup analysis was performed by race, sex, and health status. The race was divided into Caucasian, Asian, American and brown race individuals. Healthy status was divided into CAD, type 2 diabetes mellitus (T2DM), hypertension, obesity, and healthy individuals. In some studies, subjects were divided into multiple subpopulations (e.g. individuals with different diseases or individuals from different races, etc.). Each subpopulation was regarded as an independent comparison in this study.

### 2.6 Evaluation of heterogeneity

Heterogeneity was tested by the *I* ^2^ statistic and Cochran's χ2‐based Q statistic. In order to enhance the credibility of the analysis data, all results were recalculated after excluding studies with heterogeneity in the analysis of the impacts of rs1800795 on cardiometabolic risk factors. In contrast, a random‐effects model (DerSimonian‐Laird method) was used to analyse the impact of rs1800795 on PCAD.^27^

### 2.7 Publication bias test

The Begg funnel plot and Egger linear test evaluated the probability of publication bias among the included studies.^28^

### 2.8 Risk bias test

The risk bias among the included studies was evaluated by the risk‐of‐bias plot,^29^ in which different colours represent different levels of risk bias. For instance, green indicates a low risk bias, while red suggests a high risk bias.

### 2.9 The primary and secondary results in this meta‐analysis

#### 2.9.1 Primary results

1. The impacts of rs1800795 on TG, TC, LDL‐C, HDL‐C, FPG, SBP, DBP, BMI or WC in an integrated population (i.e. Caucasian, Asian, American and brown race individuals).
2. The impact of rs1800795 on PCAD risk in allelic model (*C* vs. *G*), additive model (*CC* vs. *GG*), heterozygote model (*GC* vs. *GG*), dominant model (*GC* + *CC* vs. *GG*), recessive model (*CC* vs. *GG* + *GC*) and overdominant model (*GC* vs. *GG* + *CC*).

#### 2.9.2 Secondary results

1. The impacts of rs1800795 on TG, TC, LDL‐C, HDL‐C, FPG, SBP, DBP, BMI or WC in specific population, including Caucasians, Asians, Americans, brown race individuals, males, females, CAD patients, T2DM patients, hypertension patients, obesity patients and healthy individuals.
2. The impact of rs1800795 on PCAD risk in brown race population under different genetic models.

## 3 RESULTS

### 3.1 Study selection and characteristics

**FIGURE 1:**
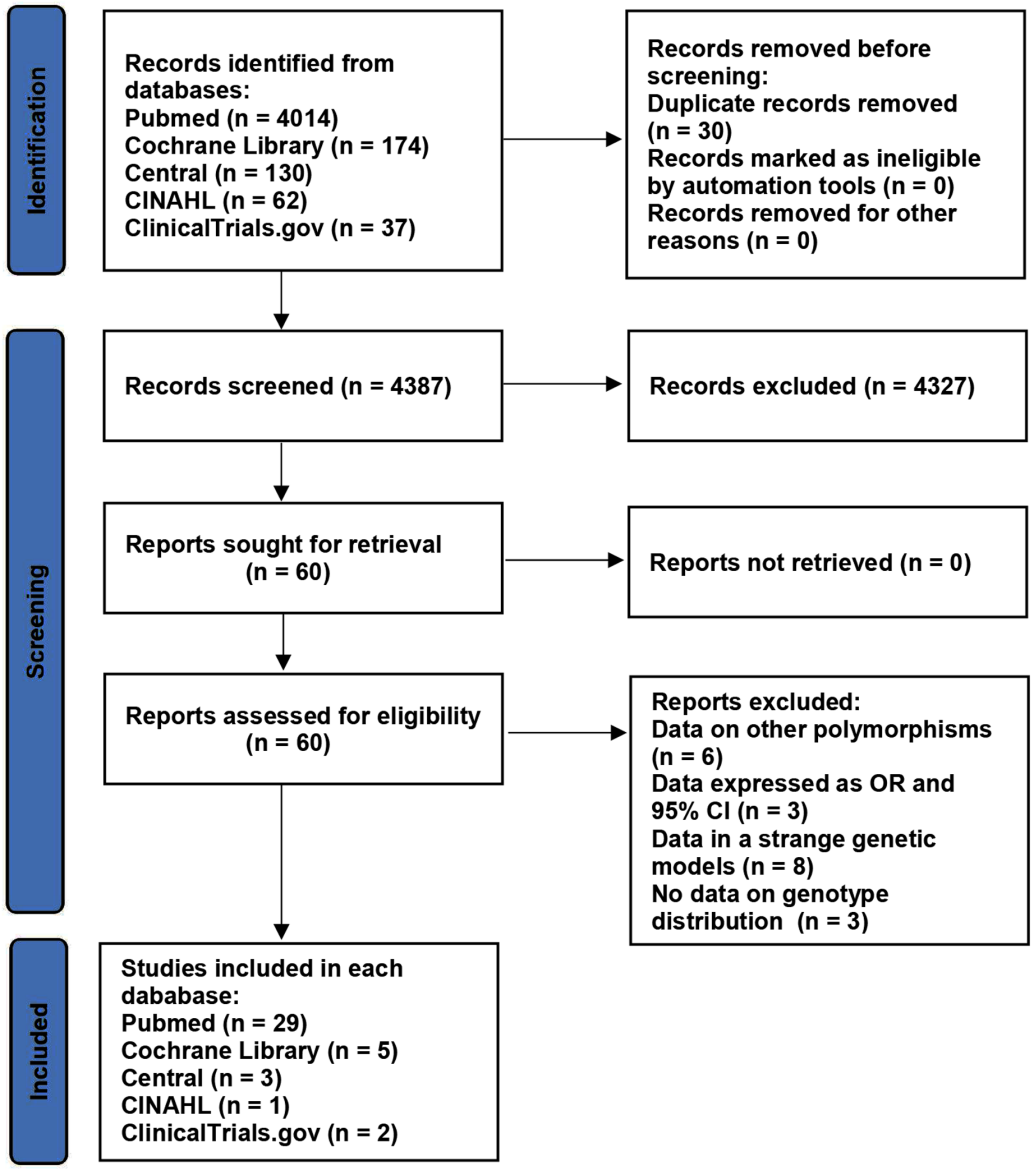
Flow diagram of the studies selection process.

The details of the study selection are summarized in Figure 1. The present study included 40 literatures in a total of 26,543 individuals, including 1328 patients with PCAD (see Table S4 for more details).

### 3.2 Impact of rs1800795 on lipid profile

**FIGURE 2:**
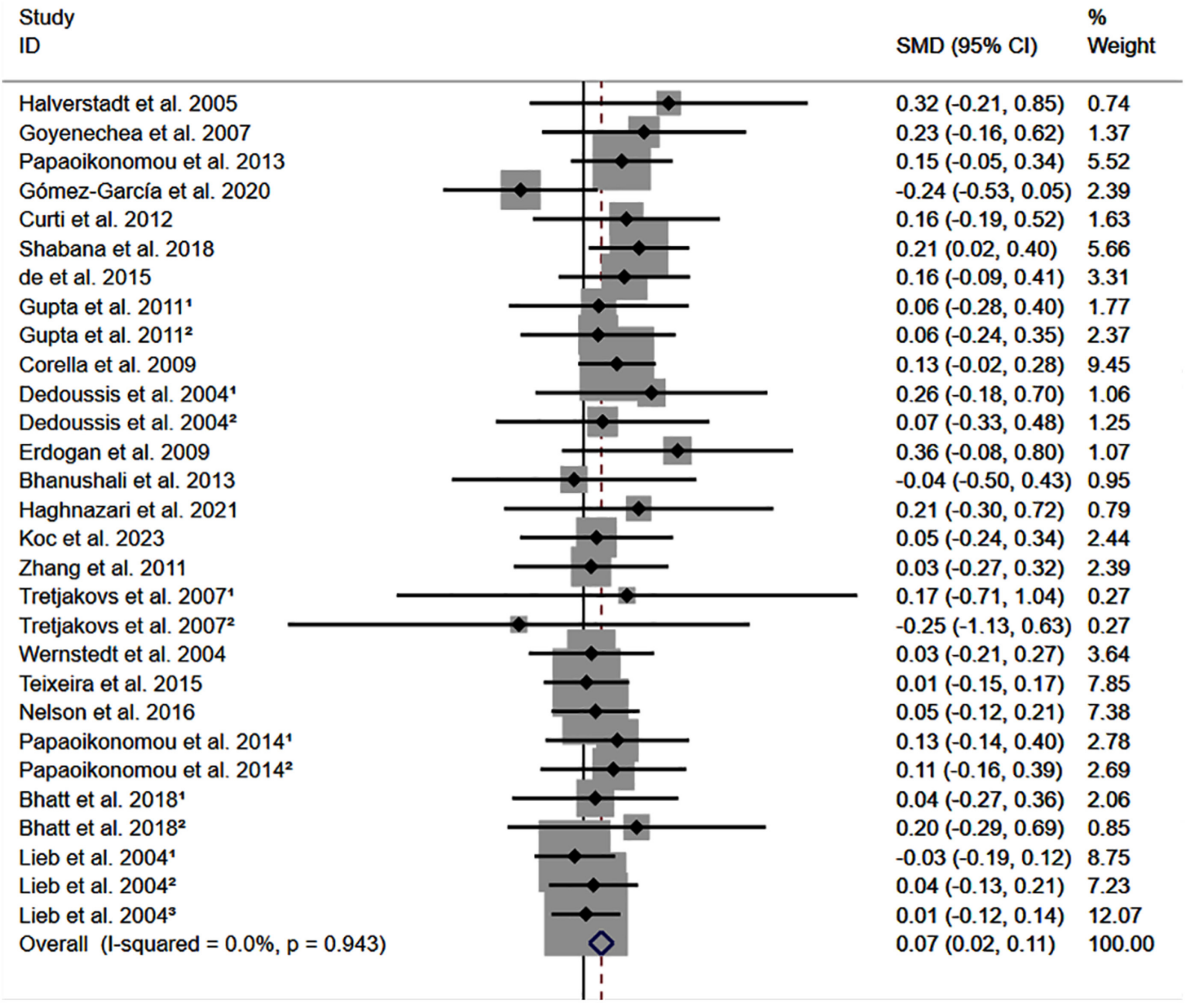
Forest plot of the meta‐analysis between rs1800795 and low‐density lipoprotein cholesterol levels.

**FIGURE 3:**
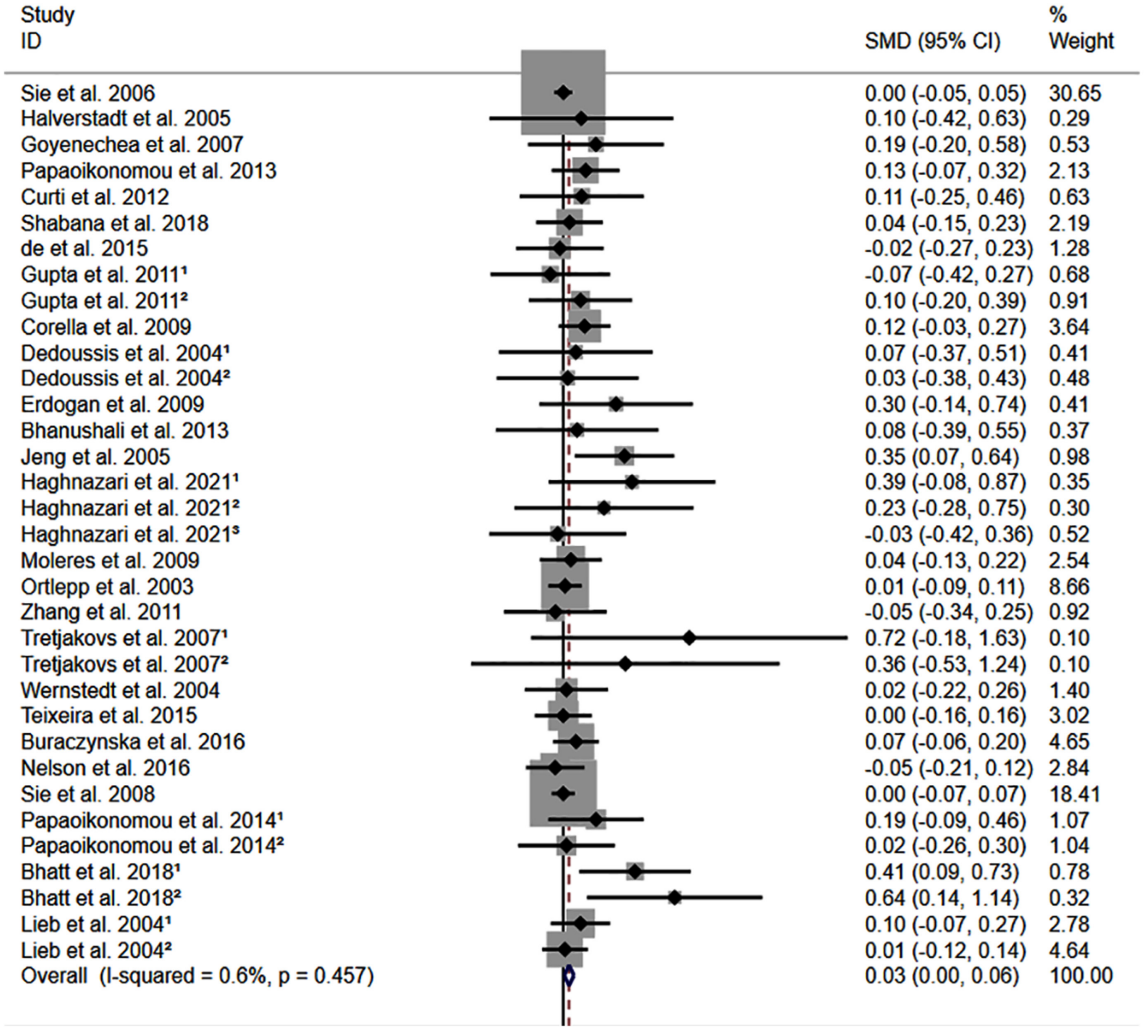
Forest plot of the meta‐analysis between rs1800795 and total cholesterol levels.

All the results stated below were the data excluded heterogeneity. rs1800795 C allele was linked to an increased LDL‐C (Figure 2) and TC (Figure 3) and decreased HDL‐C (Figure S1) levels. However, no significant impact was observed of rs1800795 with TG levels (Figure S2). Subgroup analysis indicated that the impacts of rs1800795 on LDL‐C and TC levels were significant in Caucasian and brown‐race individuals (Table 1). In contrast, the impact of rs1800795 on HDL‐C levels was significant in T2DM patients, hypertension patients, and brown‐race individuals (Table 1). In addition, the impact of rs1800795 on TG levels was significant in hypertension patients, obesity patients and brown‐race individuals (Table 1).

**TABLE 1:** Meta‐analysis of the IL‐6 rs1800795 variant with cardiometabolic risk factors.

Groups or subgroups	Subjects	*P*_*H*_	SMD (95% CI)	*P*_SMD_	Groups or subgroups	Subjects	*P*_*H*_	SMD (95% CI)	*P*_SMD_
*Overall results*					*Recalculated results that eliminated heterogeneity*				
TG					TG				
All	9054	<0.001	0.03 (−0.05 to 0.12)	0.43	All	7193	0.16	0.04 (−0.01 to 0.09)	0.11
Race					Race				
Caucasian	4892	0.10	−0.02 (−0.11 to 0.08)	0.72	Caucasian	4085	0.41	0.01 (−0.06 to 0.07)	0.84
Asian	729	0.09	−0.06 (−0.40 to 0.29)	0.74	Asian	729	0.09	−0.05 (−0.26 to 0.15)	0.61
American	***667***	0.01	−0.53 (−1.32 to 0.25)	0.18	American	‐	‐	‐	‐
Brown race	2766	0.01	0.14 (0.01 to 0.26)	0.03	Brown race	2.379	0.31	0.11 (0.03 to 0.20)	0.01
Sex					Sex				
Male	***2055***	0.07	−0.03 (−0.40 to 0.34)	0.88	Male	1969	0.24	−0.01 (−0.10 to 0.09)	0.93
Female	556	0.30	−0.12 (−0.32 to 0.07)	0.23	Female	364	0.74	−0.03 (−0.24 to 0.18)	0.77
Health status					Health status				
CAD	120	0.71	0.32 (−0.10 to 0.73)	0.13	CAD	120	0.71	0.32 (−0.10 to 0.73)	0.13
T2DM	1918	0.02	0.07 (−0.17 to 0.30)	0.58	T2DM	1818	0.07	−0.03 (−0.14 to 0.09)	0.64
Hypertension	959	0.88	0.14 (0.00 to 0.27)	0.04	Hypertension	959	0.88	0.14 (0.00 to 0.27)	0.04
Obesity	509	0.01	0.10 (−0.27 to 0.48)	0.59	Obesity	317	0.78	0.29 (0.06 to 0.52)	0.01
Healthy individuals	3507	<0.001	−0.01 (−0.17 to 0.16)	0.95	Healthy individuals	3261	0.47	−0.00 (−0.07 to 0.07)	0.93
TC					TC				
All	22,610	<0.001	0.10 (0.04 to 0.15)	<0.001	All	21,479	0.46	0.03 (0.00 to 0.06)	0.02
Race					Race				
Caucasian	18,448	<0.01	0.09 (0.03 to 0.14)	<0.01	Caucasian	17,628	0.84	0.02 (0.01 to 0.03)	0.03
Asian	***729***	0.06	0.16 (−0.23 to 0.54)	0.43	Asian	729	0.06	0.16 (−0.05 to 0.36)	0.13
American	667	0.60	−0.03 (−0.19 to 0.13)	0.69	American	667	0.60	−0.03 (−0.19 to 0.13)	0.69
Brown race	2766	<0.01	0.15 (0.01 to 0.29)	0.04	Brown race	2455	0.26	0.09 (0.01 to 0.17)	0.04
Sex					Sex				
Male	2055	0.40	0.02 (−0.07 to 0.12)	0.61	Male	2055	0.40	0.02 (−0.07 to 0.12)	0.61
Female	556	0.61	0.07 (−0.11 to 0.25)	0.44	Female	556	0.61	0.07 (−0.11 to 0.25)	0.44
Health status					Health status				
CAD	1442	0.06	0.24 (0.02 to 0.47)	0.03	CAD	699	0.41	0.12 (−0.04 to 0.27)	0.15
T2DM	2740	0.17	0.02 (−0.09 to 0.14)	0.66	T2DM	2524	0.84	0.07 (−0.01 to 0.16)	0.09
Hypertension	959	0.90	0.01 (−0.13 to 0.14)	0.93	Hypertension	959	0.90	0.01 (−0.12 to 0.14)	0.93
Obesity	509	0.02	0.40 (0.04 to 0.76)	0.03	Obesity	432	0.04	0.26 (0.05 to 0.48)	0.01
Healthy individuals	14,919	0.02	0.06 (−0.01 to 0.13)	0.08	Healthy individuals	14,824	0.80	0.01 (−0.02 to 0.05)	0.44
LDL‐C					LDL‐C				
All	8695	0.03	0.11 (0.05 to 0.17)	<0.001	All	8429	0.94	0.07 (0.02 to 0.11)	<0.01
Race					Race				
Caucasian	4565	0.91	0.06 (0.00 to 0.12)	0.04	Caucasian	4565	0.91	0.06 (0.00 to 0.12)	0.04
American	667	0.32	0.07 (−0.09 to 0.23)	0.39	American	667	0.32	0.07 (−0.09 to 0.23)	0.39
Brown race	2951	<0.01	0.19 (0.06 to 0.31)	<0.01	Brown race	2685	0.61	0.08 (0.00 to 0.16)	0.05
Sex					Sex				
Male	126	0.60	0.16 (−0.20 to 0.52)	0.39	Male	126	0.60	0.16 (−0.20 to 0.52)	0.39
Female	556	0.69	0.11 (−0.07 to 0.29)	0.23	Female	556	0.69	0.11 (−0.07 to 0.29)	0.23
Health status					Health status				
CAD	1442	0.91	−0.00 (−0.11 to 0.11)	0.99	CAD	1442	0.91	−0.00 (−0.11 to 0.11)	0.99
T2DM	1650	<0.01	0.14 (−0.07 to 0.36)	0.20	T2DM	1550	0.27	0.06 (−0.05 to 0.17)	0.30
Hypertension	959	0.90	0.02 (−0.12 to 0.15)	0.81	Hypertension	959	0.90	0.02 (−0.12 to 0.15)	0.81
Obesity	432	0.86	0.08 (−0.13 to 0.29)	0.47	Obesity	432	0.86	0.08 (−0.13 to 0.28)	0.46
Healthy individuals	1986	0.35	0.12 (0.02 to 0.23)	0.02	Healthy individuals	1891	0.83	0.08 (−0.02 to 0.17)	0.11
HDL‐C					HDL‐C				
All	20,464	<0.001	−0.02 (−0.11 to 0.06)	0.58	All	8246	0.46	−0.05 (−0.09 to −0.00)	0.04
Race					Race				
Caucasian	16,519	<0.001	−0.03 (−0.11 to 0.06)	0.52	Caucasian	4567	0.83	−0.02 (−0.08 to 0.04)	0.57
American	667	0.20	0.01 (−0.30 to 0.32)	0.96	American	667	0.20	0.07 (−0.09 to 0.23)	0.42
Brown race	2766	<0.001	−0.00 (−0.24 to 0.24)	0.98	Brown race	2500	0.44	−0.13 (−0.22 to −0.05)	<0.01
Sex					Sex				
Male	126	0.24	0.01 (−0.47 to 0.49)	0.97	Male	126	0.24	−0.04 (−0.40 to 0.33)	0.85
Female	556	0.65	0.60 (−0.12 to 0.24)	0.51	Female	556	0.65	0.06 (−0.12 to 0.24)	0.51
Health status					Health status				
CAD	1442	0.11	0.03 (−0.17 to 0.22)	0.80	CAD	863	0.15	−0.04 (−0.18 to 0.10)	0.59
T2DM	2740	<0.001	0.22 (−0.15 to 0.58)	0.25	T2DM	1550	0.87	−0.13 (−0.24 to −0.02)	0.02
Hypertension	959	0.45	−0.19 (−0.32 to −0.06)	0.01	Hypertension	959	0.45	−0.19 (−0.32 to −0.06)	0.01
Obesity	509	0.39	−0.09 (−0.29 to 0.10)	0.34	Obesity	509	0.39	−0.09 (−0.28 to 0.10)	0.34
Healthy individuals	12,773	<0.001	−0.03 (−0.15 to 0.09)	0.58	Healthy individuals	2395	0.76	0.00 (−0.08 to 0.08)	0.93
Fasting plasma glucose					Fasting plasma glucose				
All	3809	0.44	0.09 (0.02 to 0.15)	0.01	All	3809	0.44	0.09 (0.02 to 0.15)	0.01
Race					Race				
Caucasian	1436	0.65	0.04 (−0.07 to 0.14)	0.52	Caucasian	1436	0.65	0.04 (−0.07 to 0.14)	0.52
Brown race	1554	0.26	0.15 (0.04 to 0.25)	0.01	Brown race	1554	0.26	0.15 (0.04 to 0.25)	0.01
Sex					Sex				
Female	370	0.55	−0.00 (−0.22 to 0.22)	0.99	Female	370	0.55	−0.00 (−0.22 to 0.22)	0.99
Health status					Health status				
T2DM	308	0.10	0.14 (−0.08 to 0.36)	0.22	T2DM	308	0.10	0.14 (−0.08 to 0.36)	0.22
Hypertension	959	0.48	0.04 (−0.09 to 0.18)	0.54	Hypertension	959	0.48	0.04 (−0.09 to 0.18)	0.54
Obesity	432	0.43	0.18 (−0.03 to 0.39)	0.09	Obesity	432	0.43	0.18 (−0.03 to 0.39)	0.09
Healthy individuals	1437	0.20	0.10 (−0.01 to 0.21)	0.08	Healthy individuals	1437	0.20	0.10 (−0.01 to 0.21)	0.08
Systolic blood pressure					Systolic blood pressure				
All	16,834	0.67	0.01 (−0.02 to 0.04)	0.48	All	16,834	0.67	0.01 (−0.02 to 0.04)	0.48
Race					Race				
Caucasian	15,010	0.43	0.00 (−0.03 to 0.03)	0.91	Caucasian	15,010	0.43	0.00 (−0.03 to 0.03)	0.91
Brown race	1312	0.99	0.12 (0.01 to 0.24)	0.04	Brown race	1312	0.99	0.12 (0.01 to 0.24)	0.04
Sex					Sex				
Female	370	0.77	0.10 (−0.12 to 0.32)	0.38	Female	370	0.77	0.10 (−0.12 to 0.32)	0.38
Health status					Health status				
CAD	1322	0.61	−0.03 (−0.14 to 0.09)	0.64	CAD	1322	0.61	−0.03 (−0.14 to 0.09)	0.64
Hypertension	959	0.66	0.13 (0.00 to 0.26)	0.05	Hypertension	959	0.66	0.13 (0.00 to 0.26)	0.05
Obesity	522	0.99	0.08 (−0.11 to 0.27)	0.42	Obesity	522	0.99	0.08 (−0.11 to 0.27)	0.42
Healthy individuals	13,519	0.25	0.01 (−0.03 to 0.04)	0.80	Healthy individuals	13,519	0.25	0.01 (−0.03 to 0.04)	0.80
Diastolic blood pressure					Diastolic blood pressure				
All	9758	0.70	0.02 (−0.03 to 0.06)	0.48	All	9758	0.70	0.02 (−0.03 to 0.06)	0.48
Race					Race				
Caucasian	8229	0.66	−0.00 (−0.04 to 0.04)	0.96	Caucasian	8229	0.66	−0.00 (−0.04 to 0.04)	0.96
Brown race	1312	0.76	0.10 (−0.01 to 0.21)	0.09	Brown race	1312	0.76	0.10 (−0.01 to 0.21)	0.09
Sex					Sex				
Female	370	0.46	0.11 (−0.12 to 0.33)	0.36	Female	370	0.46	0.11 (−0.12 to 0.33)	0.36
Health status					Health status				
CAD	1322	0.65	−0.00 (−0.11 to 0.11)	0.97	CAD	1322	0.65	−0.00 (−0.11 to 0.11)	0.97
Obesity	522	0.56	0.07 (−0.12 to 0.26)	0.47	Obesity	522	0.56	0.07 (−0.12 to 0.26)	0.47
Healthy individuals	7302	0.58	0.00 (−0.05 to 0.05)	0.89	Healthy individuals	7302	0.58	0.00 (−0.05 to 0.05)	0.89
Body mass index					Body mass index				
All	19,126	<0.001	0.25 (0.15 to 0.35)	<0.001	All	18,774	0.22	0.05 (0.02 to 0.08)	<0.01
Race					Race				
Caucasian	16,575	0.06	0.04 (−0.02 to 0.09)	0.16	Caucasian	16,555	0.28	0.04 (0.00 to 0.07)	0.03
Brown race	1732	<0.001	0.76 (0.35 to 1.16)	<0.001	Brown race	1400	0.73	0.20 (0.09 to 0.32)	<0.001
Sex					Sex				
Male	2326	0.01	0.26 (−0.03 to 0.55)	0.08	Male	2306	0.13	0.08 (−0.01 to 0.17)	0.08
Female	468	0.42	0.09 (−0.11 to 0.28)	0.39	Female	468	0.42	0.09 (−0.11 to 0.28)	0.39
Health status					Health status				
CAD	1342	0.94	−0.02 (−0.13 to 0.09)	0.73	CAD	1342	0.93	−0.02 (−0.13 to 0.09)	0.73
T2DM	719	<0.001	0.56 (−0.19 to 1.31)	0.14	T2DM	619	0.53	0.06 (−0.10 to 0.22)	0.47
Obesity	509	0.66	0.29 (0.10 to 0.48)	<0.01	Obesity	509	0.66	0.29 (0.10 to 0.48)	<0.01
Healthy individuals	14,461	<0.001	0.24 (0.10 to 0.38)	<0.01	Healthy individuals	14,280	0.36	0.04 (0.01 to 0.08)	0.02
Waist circumference					Waist circumference				
All	2647	0.31	0.15 (0.07 to 0.23)	<0.001	All	2647	0.31	0.15 (0.07 to 0.23)	<0.001
Race					Race				
Caucasian	1292	0.07	0.09 (−0.02 to 0.20)	0.11	Caucasian	1292	0.07	0.09 (−0.02 to 0.20)	0.11
Brown race	1355	0.89	0.20 (0.09 to 0.31)	<0.001	Brown race	1355	0.89	0.20 (0.09 to 0.31)	<0.001
Sex					Sex				
Female	370	0.92	0.24 (0.02 to 0.46)	0.04	Female	370	0.92	0.24 (0.02 to 0.46)	0.04
Health status					Health status				
Obesity	432	0.51	0.24 (0.03 to 0.45)	0.03	Obesity	432	0.51	0.24 (0.03 to 0.45)	0.03
Healthy individuals	1059	0.07	0.14 (0.02 to 0.27)	0.03	Healthy individuals	1059	0.07	0.14 (0.02 to 0.27)	0.03

Abbreviations: 95% CI, 95% confidence interval; CAD, coronary artery disease; HDL‐C, high‐density lipoprotein cholesterol; IL‐6, interleukin‐6 gene; LDL‐C, low‐density lipoprotein cholesterol; *P*_H_, P for heterogeneity; SMD, standardized mean difference; T2DM, type 2 diabetes mellitus; TC, total cholesterol; TG, triglycerides.

### 3.3 Impacts of rs1800795 on other cardiometabolic risk factors

**FIGURE 4:**
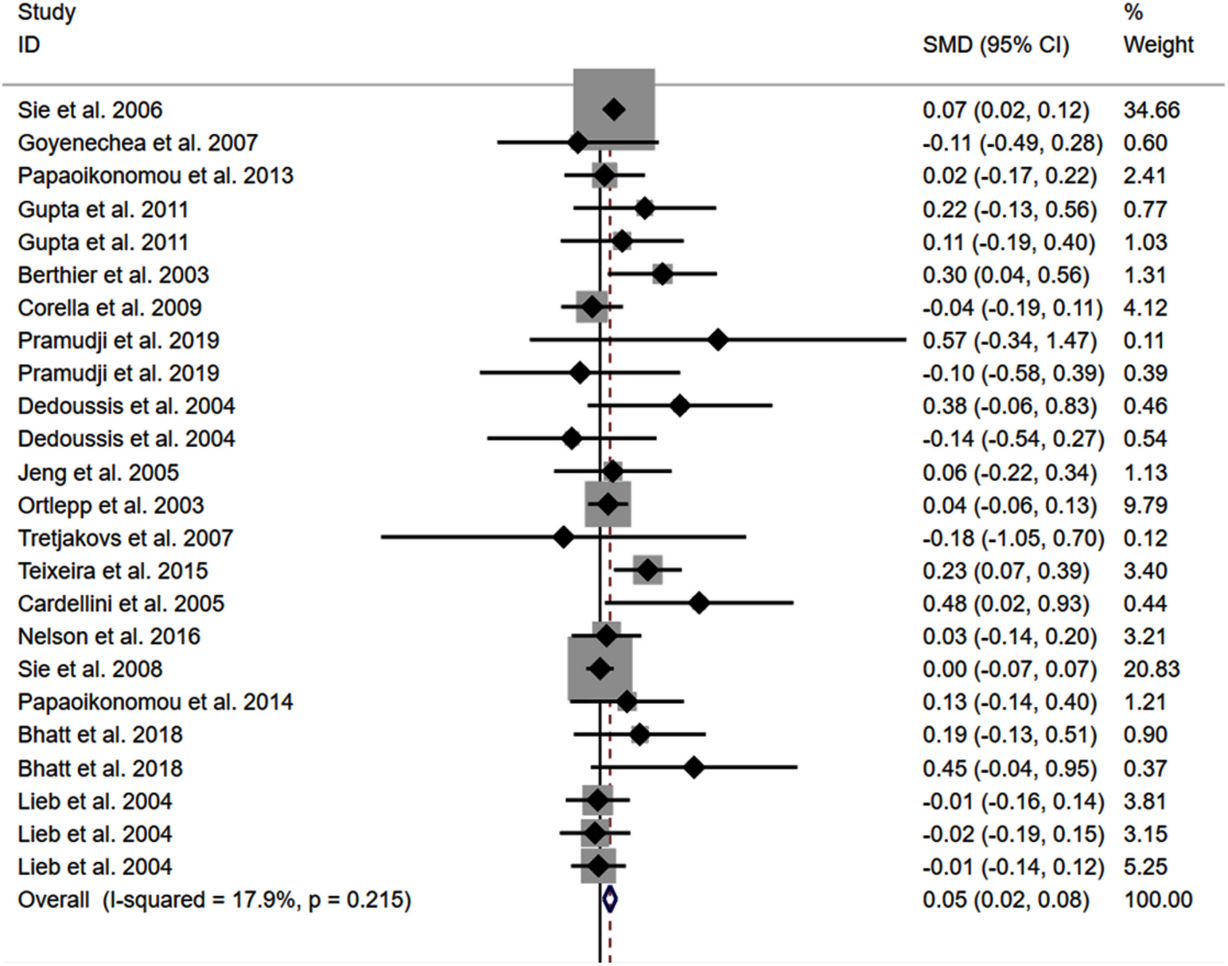
Forest plot of the meta‐analysis between rs1800795 and body mass index.

The consistent findings for rs1800795 on other cardiometabolic risk factors were the increase in FPG (Figure S3), BMI (Figure 4), and WC (Figure S4). However, no significant effect was observed of rs1800795 with SBP (Figure S5) and DBP (Figure S6). Subgroup analysis indicated that the impact of rs1800795 on FPG was significant in brown‐race individuals (Table 1). In contrast, the impact of rs1800795 on BMI was significant in Caucasian individuals, brown‐race individuals, healthy individuals, and obese patients (Table 1). In addition, the impact of rs1800795 on WC was significant in brown‐race individuals, female individuals, healthy individuals, and obese patients (Table 1). However, the impact of rs1800795 on diastolic blood pressure was not statistically significant (Table 1).

### 3.4 Impact of rs1800795 on the risk of PCAD

**FIGURE 5:**
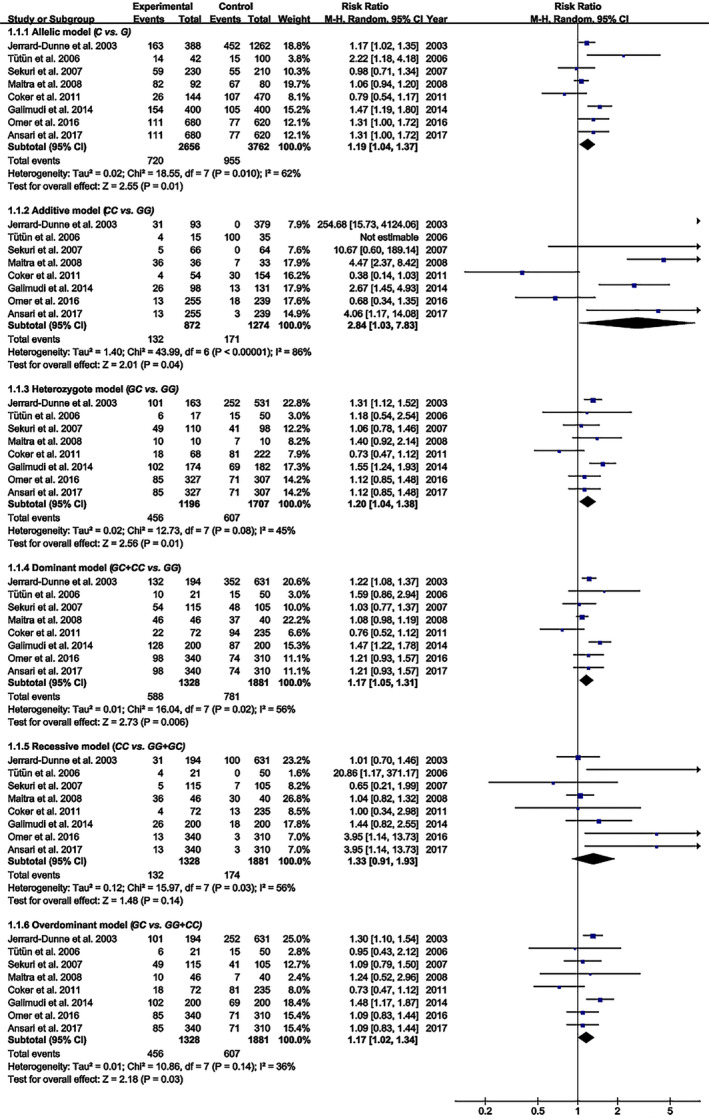
Forest plot of the meta‐analysis between rs1800795 and premature coronary artery disease.

The C allele of rs1800795 was linked to an increased risk of PCAD in the allelic, additive, heterozygote, dominant, and overdominant models (Figure 5; Table 2). Subgroup analysis indicated that the impact of rs1800795 on PCAD was significant in brown‐race individuals (Table 2).

**TABLE 2:** Meta‐analysis of the IL‐6 rs1800795 variant with premature coronary artery disease.

Groups or subgroups	Subjects	RR (95% CI)	*P*_RR_
Allelic model (***C* vs. *G***)			
All	3209	1.19 (1.04–1.37)	0.01
		04	
Brown race	2384	1.06 (1.01–1.12)	0.03
		04	
Additive model (***CC* vs. *GG***)			
All	3209	2.84 (1.03–7.83)	0.04
		04	
Brown race	2384	1.04 (1.00–1.09)	0.05
		04	
Heterozygote model (***GC* vs. *GG***)			
All	3209	1.20 (1.04–1.38)	0.01
Brown race	2384	1.17 (1.10–1.24)	0.02
Dominant model (GC ***+ CC* vs. *GG***)			
All	3209	1.17 (1.05–1.31)	0.01
		04	
Brown race	2384	1.14 (1.00–1.30)	0.04
		04	
Recessive model (***CC* vs. *GG + GC***)			
All	3209	1.33 (0.91–1.93)	0.14
		04	
Brown race	2384	1.03 (1.01–1.05)	0.01
Overdominant model (***GC* vs. *GG + CC***)			
All	3209	1.17 (1.02–1.34)	0.03
Brown race	2384	1.10 (1.01–1.19)	0.04

Abbreviations: 95% CI, 95% confidence interval; *IL‐6*, interleukin‐6 gene; RR, risk ratios.

### 3.5 Evaluation of heterogeneity

Significant heterogeneity was detected among rs1800795, lipid and BMI (Table 1). Notably, the recalculated results remained relatively the same after excluding the studies with heterogeneity (Table 1), indicating the robustness of the calculated results.

### 3.6 Publication bias test

Begg funnel plot was used to evaluate publication bias among the included studies. This meta‐analysis had no publication bias, which was confirmed by the Egger linear regression test.

### 3.7 Risk bias test

In analysis the risk bias of rs1800795 with LDL‐C (Figure S7), 86.4% (19/22) of studies presented with green colour (Figure S7), indicating a low risk bias. Since the included studies for the bias test of LDL‐C were largely consistent with other lipids or cardiometabolic parameters (see Table S4 for more details), it indicated that the studies included for the identification of cardiometabolic risk factors were of relatively high quality. Additionally, in analysis the risk bias of rs1800795 with PCAD (Figure S8), 87.5% (7/8) of studies presented with green colour (Figure S8), suggesting a low risk bias. In summary, the results calculated in this paper were quite reliable due to a low risk bias.

## 4 DISCUSSION

The present study indicated that the rs1800795 C allele was linked to an increased risk of PCAD (Figure 5, Table 2) and elevation levels of LDL‐C (Figure 2; Table 1), HDL‐C (Figure S1; Table 1), TC (Figure 3; Table 1), FPG (Figure S3; Table 1), BMI (Figure 4, Table 1), and WC (Figure S4; Table 1). It indicated that the impact of rs1800795 on PCAD was mediated, at least partly, by the impacts of rs1800795 on cardiometabolic risk factors.

The impact of rs1800795 on PCAD was significant in the brown race population (Table 2). Since the impacts of rs1800795 on cardiometabolic risk factors were widely significant in the brown race population (i.e. Mexican, Brazilian, Pakistani, Indian, Iranian, and Turkish) (Table 1), it indicated that the cardiometabolic disorder mediated the impact of rs1800795 on PCAD in brown race population.

According to the 2018 American College of Cardiology (ACC)/American Heart Association (AHA),^30^ the 2019 European Society of Cardiology (ESC)/European Atherosclerosis Society (EAS),^31^ and the adult treatment panel III (ATP III) cholesterol guidelines,^32^ LDL‐C was considered the major cause of CAD and treated as the primary target for therapy, while other lipids were used as the secondary or supplementary therapeutic targets. In the present study, the rs1800795 C allele was linked to increased LDL‐C and TC levels in Caucasians (Table 1), indicating that Caucasians with the rs1800795 C allele were at high risk of PCAD.

One particular reason could be proposed to interpret why the impacts of rs1800795 on cardiometabolic risk factors were primarily significant in the brown race population and Caucasians, but not Asians. That is, the integrated brown race population [ie, Mexican (11.5%–16.5%), Brazilian (16.5%–27.8%), Pakistani (72%–80%), Indian (12.5%–32.4%), Iranian (14.3%–36.3%), and Turkish (25.5%–26.4%)] and Caucasians had a much higher carrying rate of rs1800795 C allele than Asians (brown race population: average: 45.75%, range from 11.5%–80%; Caucasians: average: 40.4%, range from 26.2%–54.6%; Asians: average: 0.15%, range from 0%–0.3%).^33^, ^34^ Since the C allele of rs1800795 largely determined serum IL‐6 levels^8^, ^17^, ^18^ and thus linked to CAD^3^, ^4^, ^5^ and atherosclerosis,^7^, ^8^, ^9^, ^10^, ^11^, ^12^ an increasing number of C allele will no doubt disturb cardiometabolic and increase the risk of PCAD. This can be used to explain the presenting findings. However, only one study^35^ has investigated the impact of rs1800795 on PCAD in Caucasians. Future Caucasian‐based clinical trials are certainly needed.

The carrying rate of the C allele in Americans was as high as 44.35% (range from 35.3% to 53.4%),^33^, ^34^ indicating that Americans with rs1800795 had an increased risk of PCAD. However, the impacts of rs1800795 on cardiometabolic risk factors in Americans did not show statistically significant, possibly due to low statistical power (2 comparisons with 667 individuals) (Table 1), which lowered the credibility and needs to be uncovered by future large‐scale American‐based studies.

The strongest impacts of rs1800795 on cardiometabolic risk factors were in obese patients (Table 1), whose SMD values were much larger than those calculated in other subpopulations (Table 1). It indicated that obese patients with rs1800795 had a very high risk of PCAD. Interestingly, this hypothesis was partially verified in the present study in which the rs1800795 C allele was linked to an increased BMI and WC in obese patients (Table 1). These metabolic disorders will no doubt promote the onset of PCAD, according to Stone et al.^16^ study.

It was not until 2017 that the proof of principle for the inflammation hypothesis of atherothrombosis was provided in the 10,000‐participant Canakinumab Anti‐Inflammatory Thrombosis Outcomes Study (CANTOS), which demonstrated that targeting IL‐1β using canakinumab lowered major cardiovascular events in the absence of any effect on cholesterol and blood pressure.^36^ In addition, canakinumab also inhibited the IL‐6 signalling pathway independent of lipid‐level lowering.^10^ In contrast, a Phase 2 clinical trial (RESCUE) demonstrated that targeting IL‐6 ligand using ziltivekimab inhibited atherosclerosis progress by reducing high‐sensitivity C‐reactive protein (CRP), fibrinogen, serum amyloid A, haptoglobin, secretory phospholipase A2, and lipoprotein(a), independent of TC/HDL‐C ratio.^8^ Together, IL‐6 inhibition with ziltivekimab or canakinumab may prevent atherosclerosis and/or CAD independent of lipid levels and blood pressure.

Since the impact of rs1800795 on PCAD may be essentially attributed to high levels or activity of IL‐6, IL‐6 inhibition with ziltivekimab or canakinumab may benefit high‐risk populations (e.g. brown race population, Caucasians and obese patients etc.) with rs1800795 to prevent PCAD. Since ziltivekimab or canakinumab appear not to inhibit cardiometabolic risk factors (e.g. lipid and blood pressure),^8^, ^10^, ^36^ ziltivekimab or canakinumab in combination with other medications (e.g. antihypertensive drugs, hypoglycemic drugs, and statins etc.) may be needed for high‐risk populations with specific illness (e.g. hypertension, T2DM, and dyslipidemia, etc.) in the process of PCAD prevention. However, these hypotheses must be tested strictly before being used in clinical practice.

The present meta‐analysis has several strengths: (I) this is the first meta‐analysis that systematically investigated the impacts of rs1800795 on cardiometabolic profile and PCAD; (II) all results were recalculated after eliminated studies with heterogeneity in the association analysis between rs1800795 and cardiometabolic risk factors, which advances the preciseness of conclusions drawn in this paper; (III) the present data indicate that genetic screening of the rs1800795 variant in high‐risk populations (e.g. brown race population, Caucasians, and obese patients, etc.) is meaningful for PCAD prevention. Meanwhile, several serious limitations should be noted when interpretation of the results: (I) the interactions of rs1800795 with other variant locus or environmental factors on cardiometabolic risk factors and PCAD have yet to be investigated in the present meta‐analysis due to the lack of original data from the included studies. In other words, more precise results could have been gained if more detailed individual data were available or the stratification analyses based on environmental factors, such as diet, exercise, smoking, etc. were performed; (II) a random‐effects model was adopted to analysis the association between rs1800795 and PCAD, significant heterogeneity may encounter perhaps due to various, center settings, populations enrolled etc. Therefore, calling for cautious interpretation of the results of rs1800795 with PCAD; (III) the impacts of rs1800795 on cardiometabolic parameters in many occasions (e.g. in obese patients and females) were assessed by very few studies; thus, the evidence to support it is low.

## AUTHOR CONTRIBUTIONS

**Yang Liu:** Formal analysis (equal); visualization (equal); writing – original draft (equal). **Yuan Chen:** Data curation (equal); formal analysis (equal); investigation (equal); writing – review and editing (equal). **Yi Lin:** Data curation (equal); formal analysis (equal); investigation (equal); writing – original draft (equal). **Baozhu Wei:** Formal analysis (equal); supervision (equal); writing – original draft (equal). **Zhi Luo:** Conceptualization (equal); supervision (equal); writing – review and editing (equal).

## FUNDING INFORMATION

No funding was received for conducting this study.

## CONFLICT OF INTEREST STATEMENT

The authors declare no competing interests.

## Supporting information


Data S1.

## Data Availability

The data that supports the findings of this study are available in the supplementary material of this article.
