# Supplementary material for: Impacts of pro‐inflammatory cytokines variant on cardiometabolic profile and premature coronary artery disease: A systematic review and meta‐analysis
Source: J Cell Mol Med. 2024 Apr 17;28(8):e18311. doi: 10.1111/jcmm.18311 (PMC11024509; doi:10.1111/jcmm.18311)
Supplement: Supplementary file 1 — Data S1. [file JCMM-28-e18311-s001.doc]

**Supplementary Material**

**Supplemental Tables**

*Table S1* **Checklist of items to include when reporting a systematic review or meta-analysis.**

*Table S2* **PICOS criteria for inclusion of studies.**

*Table S3* **PubMed search strategy.**

*Table S4* **Characteristics of the included studies.**

**Supplemental Figures**

*Figure S1* **Forest plot of the meta-analysis between rs1800795 and high-density lipoprotein cholesterol levels.**

*Figure S2* **Forest plot of the meta-analysis between rs1800795 and triglycerides levels.**

*Figure S3* **Forest plot of the meta-analysis between rs1800795 and fasting plasma glucose levels.**

*Figure S4* **Forest plot of the meta-analysis between rs1800795 and waist circumference.**

*Figure S5* **Forest plot of the meta-analysis between rs1800795 and systolic blood pressure.**

*Figure S6* **Forest plot of the meta-analysis between rs1800795 and diastolic blood pressure.**

*Figure S7* **Risk bias plot of the meta-analysis between rs1800795 and low-density lipoprotein cholesterol levels.**

*Figure S8* **Risk bias plot of the meta-analysis between rs1800795 and premature coronary artery disease.**

***Table S1* Checklist of items to include when reporting a systematic review or meta-analysis.**

| **Section and Topic** | **Item #** | **Checklist item** | **Location where item is reported** |
| --- | --- | --- | --- |
| **TITLE** | | |  |
| Title | 1 | Identify the report as a systematic review. | 1 |
| **ABSTRACT** | | |  |
| Abstract | 2 | See the PRISMA 2020 for Abstracts checklist. | 2 |
| **INTRODUCTION** | | |  |
| Rationale | 3 | Describe the rationale for the review in the context of existing knowledge. | 3-5 |
| Objectives | 4 | Provide an explicit statement of the objective(s) or question(s) the review addresses. | 5 |
| **METHODS** | | |  |
| Eligibility criteria | 5 | Specify the inclusion and exclusion criteria for the review and how studies were grouped for the syntheses. | 6 |
| Information sources | 6 | Specify all databases, registers, websites, organisations, reference lists and other sources searched or consulted to identify studies. Specify the date when each source was last searched or consulted. | 6 |
| Search strategy | 7 | Present the full search strategies for all databases, registers and websites, including any filters and limits used. | 6 |
| Selection process | 8 | Specify the methods used to decide whether a study met the inclusion criteria of the review, including how many reviewers screened each record and each report retrieved, whether they worked independently, and if applicable, details of automation tools used in the process. | 6 |
| Data collection process | 9 | Specify the methods used to collect data from reports, including how many reviewers collected data from each report, whether they worked independently, any processes for obtaining or confirming data from study investigators, and if applicable, details of automation tools used in the process. | 6 |
| Data items | 10a | List and define all outcomes for which data were sought. Specify whether all results that were compatible with each outcome domain in each study were sought (e.g. for all measures, time points, analyses), and if not, the methods used to decide which results to collect. | 6 |
|  | 10b | List and define all other variables for which data were sought (e.g. participant and intervention characteristics, funding sources). Describe any assumptions made about any missing or unclear information. | 6 |
| Study risk of bias assessment | 11 | Specify the methods used to assess risk of bias in the included studies, including details of the tool(s) used, how many reviewers assessed each study and whether they worked independently, and if applicable, details of automation tools used in the process. | 9 |
| Effect measures | 12 | Specify for each outcome the effect measure(s) (e.g. risk ratio, mean difference) used in the synthesis or presentation of results. | 7 |
| Synthesis methods | 13a | Describe the processes used to decide which studies were eligible for each synthesis (e.g. tabulating the study intervention characteristics and comparing against the planned groups for each synthesis (item #5)). | 7 |
|  | 13b | Describe any methods required to prepare the data for presentation or synthesis, such as handling of missing summary statistics, or data conversions. | 7 |
|  | 13c | Describe any methods used to tabulate or visually display results of individual studies and syntheses. | 7 |
|  | 13d | Describe any methods used to synthesize results and provide a rationale for the choice(s). If meta-analysis was performed, describe the model(s), method(s) to identify the presence and extent of statistical heterogeneity, and software package(s) used. | 7 |
|  | 13e | Describe any methods used to explore possible causes of heterogeneity among study results (e.g. subgroup analysis, meta-regression). | 8 |
|  | 13f | Describe any sensitivity analyses conducted to assess robustness of the synthesized results. | 8 |
| Reporting bias assessment | 14 | Describe any methods used to assess risk of bias due to missing results in a synthesis (arising from reporting biases). | 8 |
| Certainty assessment | 15 | Describe any methods used to assess certainty (or confidence) in the body of evidence for an outcome. | 8 |
| **RESULTS** | | |  |
| Study selection | 16a | Describe the results of the search and selection process, from the number of records identified in the search to the number of studies included in the review, ideally using a flow diagram. | 10 |
|  | 16b | Cite studies that might appear to meet the inclusion criteria, but which were excluded, and explain why they were excluded. | 10 |
| Study characteristics | 17 | Cite each included study and present its characteristics. | 10 |
| Risk of bias in studies | 18 | Present assessments of risk of bias for each included study. | 11 |
| Results of individual studies | 19 | For all outcomes, present, for each study: (a) summary statistics for each group (where appropriate) and (b) an effect estimate and its precision (e.g. confidence/credible interval), ideally using structured tables or plots. | 10,11 |
| Results of syntheses | 20a | For each synthesis, briefly summarise the characteristics and risk of bias among contributing studies. | 10,11 |
|  | 20b | Present results of all statistical syntheses conducted. If meta-analysis was done, present for each the summary estimate and its precision (e.g. confidence/credible interval) and measures of statistical heterogeneity. If comparing groups, describe the direction of the effect. | 10,11 |
|  | 20c | Present results of all investigations of possible causes of heterogeneity among study results. | 11 |
|  | 20d | Present results of all sensitivity analyses conducted to assess the robustness of the synthesized results. | 11 |
| Reporting biases | 21 | Present assessments of risk of bias due to missing results (arising from reporting biases) for each synthesis assessed. | 11 |
| Certainty of evidence | 22 | Present assessments of certainty (or confidence) in the body of evidence for each outcome assessed. | 11 |
| **DISCUSSION** | | |  |
| Discussion | 23a | Provide a general interpretation of the results in the context of other evidence. | 12 |
|  | 23b | Discuss any limitations of the evidence included in the review. | 15 |
|  | 23c | Discuss any limitations of the review processes used. | 15 |
|  | 23d | Discuss implications of the results for practice, policy, and future research. | 15 |
| **OTHER INFORMATION** | | |  |
| Registration and protocol | 24a | Provide registration information for the review, including register name and registration number, or state that the review was not registered. | - |
|  | 24b | Indicate where the review protocol can be accessed, or state that a protocol was not prepared. | - |
|  | 24c | Describe and explain any amendments to information provided at registration or in the protocol. | - |
| Support | 25 | Describe sources of financial or non-financial support for the review, and the role of the funders or sponsors in the review. | 16 |
| Competing interests | 26 | Declare any competing interests of review authors. | 16 |
| Availability of data, code and other materials | 27 | Report which of the following are publicly available and where they can be found: template data collection forms; data extracted from included studies; data used for all analyses; analytic code; any other materials used in the review. | 16 |

***Table S2* PICOS criteria for inclusion of studies.**

| Parameter | Study selection criteria |
| --- | --- |
| Population | Healthy individuals or patients in specific ethnicities (eg, Caucasian, Asian, African, and American etc.) |
| Intervention or exposure | No special interventions, especially currently or in the past 1 month with a drug use history of lipid-lowering. hypoglycemic, or antihypertensive |
| Comparison | The studies compare the cardiometabolic characteristics (ie, triglycerides, total cholesterol, low-density lipoprotein cholesterol, high-density lipoprotein cholesterol, fasting plasma glucose, systolic blood pressure, diastolic blood pressure, body mass index, waist circumference) between carriers of rs1800795 C allele and carriers of rs1800795 G allele. |
| Outcomes | Cardiometabolic parameters are expressed as mean with standard deviation or standard errors, or the number of genotype in PCAD group and control group is provided, to facilitate the subsequent calculation of standardized mean difference and 95% confidence intervals, or risk ratios and 95% confidence intervals. |
| Study design | Observational study, published in English, and funded by a funding body or institution. |

***Table S3* PubMed search strategy.**

| Search | Search terms |
| --- | --- |
| **#1** | [“interleukin-6” OR “IL-6” OR “pro-inflammatory cytokines”] |
| **#2** | [“rs1800795” OR “variant” OR “polymorphism” OR “mutation” OR “variation” OR “mutant” OR “SNP” OR “single nucleotide polymorphism”] |
| **#3** | [“lipids” OR “circulating lipids” OR “blood lipids” OR “plasma lipids” OR “serum lipids” OR “lipid profile” OR “triglycerides” OR “total cholesterol” OR “low-density lipoprotein cholesterol” OR “high-density lipoprotein cholesterol” OR “TG” OR “TC” OR “LDL-C” OR “HDL-C” OR “blood pressure” OR “systolic blood pressure” OR “diastolic blood pressure” OR “FPG” OR “fasting plasma glucose” OR “FPG” OR “fasting blood glucose” OR “BMI” OR “body mass index” OR “waist circumference” OR “PCAD” OR “premature coronary artery disease” OR “PCAD” OR “early-onset coronary artery disease”] |
| **#4** | **#1** AND **#2** AND **#3** |

***Table S4*** Characteristics of the included studies.

| **First author, reference** | **Year** | **Age** | **Race** | **Sex** | **Health status** | **Number** | | **BMI, kg/m^2^** | | **TG, mmol/L** | | **TC, mmol/L** | | **LDL-C, mmol/L** | | **HDL-C, mmol/L** | | **FPG, mmol/L** | | **DBP, mmHg** | | **SBP, mmHg** | | **WC, cm** | |
| --- | --- | --- | --- | --- | --- | --- | --- | --- | --- | --- | --- | --- | --- | --- | --- | --- | --- | --- | --- | --- | --- | --- | --- | --- | --- |
|  |  |  |  |  |  | **GG** | **GC±CC** | **GG** | **GC±CC** | **GG** | **GC±CC** | **GG** | **GC±CC** | **GG** | **GG±CC** | **GG** | **GC±CC** | **GG** | **GC±CC** | **GG** | **GC±CC** | **GG** | **GC±CC** | **GG** | **GC±CC** |
| Sie MP [S1] | 2006 | 69.00±9.10 | Caucasian | M/F | Healthy subjects | 2301 | 4133 | 26.1± 3.7 | 26.35±3.75 | 1.89±0.18 | 1.72±0.17 | 6.6±1.2 | 6.6±1.2 | 3.22±0.74 | 3.46±0.74 | 1.4±0.4 | 1.3±0.4 | - | - | - | - | 139±22.3 | 139.26±22.19 | - | - |
| Halverstadt A [S2] | 2005 | 57.90±0.70 | American | M/F | Healthy subjects | 20 | 45 | - | - | - | - | 5.38±0.88 | 5.47±0.87 | 3.23±0.66 | 3.42±0.92 | 1.29±0.35 | 1.2±0.34 | - | - | - | - | - | - | - | - |
| Goyenechea E [S3] | 2007 | 34 (25-43) | Caucasian | M/F | Healthy subjects | 43 | 63 | 30.5±1.4 | 30.35±1.42 | - | - | 5.18±0.88 | 5.36±1.01 | 2.77±0.86 | 2.91±1.05 | 1.41±0.37 | 1.37±0.36 | 4.91 ± 0.34 | 5.03±0.42 | 70 ± 7 | 72.75±9.46 | 124 ± 14 | 129.75±13.83 | 93.1±8.1 | 94.96±6.91 |
| Papaoikonomou S [S4] | 2013 | 66.50±10.00 | Caucasian | M/F | Patients with T2DM | 202 | 209 | 28.92±5.16 | 29.03±4.93 | 1.25±0.49 | 1.36±0.35 | 4.78±1 | 4.92±1.17 | 4.01±1.11 | 3.75±1.01 | 1.27±0.34 | 1.23±0.35 | - | - | - | - | - | - | 99.57±12.262 | 100.02±13.09 |
| Gómez-García EF [S5] | 2020 | - | Brown race | M/F | Patients with T2DM | 153 | 63 | - | - | 1.82±0.68 | 1.71±0.78 | 6.03±1.53 | 5.51±1.48 | 3.18±1.1 | 3.35±1.01 | 1.37±0.42 | 1.27±0.26 | - | - | - | - | - | - | - | - |
| Curti ML [S6] | 2012 | 56.50±11.60 | Brown race | M/F | Patients at high cardiometabolic risk | 47 | 86 | - | - | 2.06±0.96 | 2.18±1.07 | 5.13±1.18 | 5.25±1.11 | 1.86±0.91 | 2.02± 0.6 | 1.15±0.4 | 1.08±0.27 | - | - | - | - | - | - | 93.1±8.1 | 94.96±6.91 |
| Shabana NA [S7] | 2018 | 59.10±12.60 | Brown race | M/F | Patients with premature stroke | 194 | 232 | - | - | 1.49±1.06 | 1.4±0.72 | 4.49±2.58 | 4.56±1.22 | 3.15± 0.88 | 3.28±0.8 | 1.8±1.43 | 1.73±0.49 | - | - | - | - | - | - | - | - |
| de Oliveira R [S8] | 2015 | 46.60±8.30 | Brown race | M/F | Healthy subjects | 112 | 137 | - | - | 1.55±0.49 | 1.37±0.45 | 5.3±1.09 | 5.28±0.93 | 2.37±0.67 | 2.41±0.7 | 1.45±0.49 | 1.4±0.31 | - | - | - | - | - | - | - | - |
| Gupta A [S9] | 2011 | 29.10±6.50 | Brown race | F | Patients with obesity | 42 | 150 | 28.00±3.32 | 28.82±3.92 | 1.3±0.39 | 1.25±0.49 | 4.21±0.78 | 4.15±0.81 | 2.08±0.97 | 2.13±0.85 | 1.13±0.23 | 1.11±0.22 | 5.29±0.79 | 5.35±0.77 | 83.10±8.34 | 84.8±8.65 | 125.71±14.99 | 126.53±12.72 | 86.21±9.44 | 88.71±11.47 |
| Gupta A [S9] | 2011 | 28.00±5.70 | Brown race | F | Healthy subjects | 80 | 98 | 20.69±3.43 | 21.02±2.79 | - | - | 3.83±1.04 | 3.92±0.8 | - | - | 1.16±0.28 | 1.22±0.32 | 5.12±0.87 | 5.07+0.8 | 80.44±7.97 | 80.68±6.81 | 118.39±9.48 | 119.68±10.44 | 68.71±8.64 | 70.84±8.50 |
| Berthier MT [S10] | 2003 | - | Caucasian | M | Healthy subjects | 80 | 191 | 28.44±4.46 | 29.68±3.98 | 1.66±0.80 | 1.55 ± 0.7 | - | - | 3.52±0.90 | 3.63±0.84 | - | - | 5.45±0.60 | 5.5±0.56 | - | - | - | - | 97.98±11.36 | 102.05±10.72 |
| Corella D [S11] | 2009 | 68.75±6.30 | Caucasian | M/F | Patients with CVD | 303 | 418 | 29.88±4.35 | 29.72±4.07 | - | - | 5.39±0.97 | 5.51±0.99 | - | - | 1.14±0.26 | 1.15±0.25 | - | - | - | - | - | - | - | - |
| Pramudji H [S12] | 2019 | 22.1±4.10 | Brown race | M/F | Patients with obesity and controls | 5 | 84 | 27.8±0.8 | 30.15±4.23 | - | - | - | - | - | - | - | - | - | - | - | - | - | - | - | - |
| Pramudji H [S12] | 2019 | 21.3±4.00 | Brown race | M/F | Healthy subjects | 22 | 67 | 20.7±2.0 | 20.49±2.22 | 0.97±0.32 | 0.84±0.24 | - | - | 3.11±0.56 | 3.26±0.59 | - | - | - | - | - | - | - | - | - | - |
| Dedoussis GV [S13] | 2004 | - | Caucasian | M | Healthy subjects | 31 | 55 | 19.4±2.7 | 20.7±3.7 | 1.03±0.32 | 1.04±0.35 | 4.81±0.67 | 4.86±0.7 | 3.30±0.80 | 3.35±0.61 | 1.22±0.26 | 1.19±0.16 | - | - | - | - | - | - | - | - |
| Dedoussis GV [S13] | 2004 | - | Caucasian | F | Healthy subjects | 38 | 60 | 20.8±3.3 | 20.3±3.9 | - | - | 4.97±0.93 | 4.99±0.7 | - | - | 1.19±0.21 | 1.19±0.26 | - | - | - | - | - | - | - | - |
| Guzmán-Guzmán IP [S14] | 2010 | 53(46-77) | Brown race | M/F | Patients witht obesity | 69 | 21 | - | - | 1.35±0.7 | 1.41±0.91 | - | - | 2.93±0.84 | 3.23±0.83 | - | - | - | - | 76.3±11.3 | 78.4±13.8 | 123.6±18.1 | 124.8±22.3 | - | - |
| Erdogan M [S15] | 2009 | 24.07±1.32 | Brown race | F | Patients witht PCOS | 57 | 31 | - | - | 1.61±1.05 | 1.98±0.93 | 5.05±1.09 | 5.38±1.09 | 3.18±0.78 | 3.15±0.96 | 1.48±0.38 | 1.51±0.49 | - | - | - | - | - | - | - | - |
| Bhanushali AA [S16] | 2013 | 48.00±11.00 | Brown race | M/F | Patients with CAD | 77 | 23 | - | - | 0.96±0.32 | 1±0.36 | 4.86±0.98 | 4.94±1.1 | - | - | 1.06±0.21 | 0.99±0.23 | - | - | - | - | - | - | - | - |
| Jeng JR [S17] | 2005 | 52.10±12.37 | Asian | M/F | Healthy subjects | 72 | 145 | 22.9±3.3 | 23.11±3.47 | 1.21±0.62 | 1.48±0.75 | 4.73±0.78 | 4.99±0.72 | 2.24±0.4 | 2.7±0.64 | - | - | - | - | 75±8 | 76 ±8.21 | - | - | - | - |
| Haghnazari L [S18] | 2021 | 19.65±7.33 | Brown race | M/F | Patients with T1DM | 42 | 29 | 21.56±4.39 | 27.77±4.25 | 1.5±0.67 | 1.75±0.66 | 4.3±0.83 | 4.7±1.24 | 2.65±0.36 | 2.73±0.42 | 1.25±0.2 | 1.06±0.2 | - | - | - | - | - | - | - | - |
| Haghnazari L [S18] | 2021 | 18.14±8.43 | Brown race | M/F | Healthy subjects | 44 | 22 | 22.39±3.45 | 27.11±2.12 | 1.22±0.56 | 1.59±1.08 | 4.59±0.8 | 4.78±0.84 | 2.14±0.38 | 2.58±0.63 | 1.12±0.16 | 1.09±0.2 | - | - | - | - | - | - | - | - |
| Haghnazari L [S18] | 2021 | 56.18±8.49 | Brown race | M/F | Patients with T2DM | 48 | 52 | 21.29±3.82 | 27.21±3.5 | 1.33±0.53 | 1.73±0.67 | 4.48±0.66 | 4.45±1.13 | 2.53±0.45 | 2.79±0.43 | 1.39±0.18 | 2.58±0.63 | - | - | - | - | - | - | - | - |
| Haghnazari L [S18] | 2021 | 53.84±9.14 | Brown race | M/F | Healthy subjects | 50 | 45 | 21.38±2.25 | 26.52±2.19 | 0.78±0.34 | 0.78±0.37 | 4.32±0.75 | 5.08±0.86 | - | - | 1.12±0.17 | 1.21±0.26 | - | - | - | - | - | - | - | - |
| Moleres A [S19] | 2009 | 14.55±1.06 | Caucasian | M/F | Healthy subjects | 211 | 293 | - | - | 1.13±0.73 | 1.12±0.63 | 4.2±0.62 | 4.23±0.74 | - | - | 1.41±0.29 | 1.43±0.29 | - | - | - | - | - | - | 74.2±9.34 | 74.0±8.66 |
| Ortlepp JR [S20] | 2003 | 33.00±11.00 | Caucasian | M | Healthy subjects | 611 | 1318 | 24.1±2.6 | 24.2±2.6 | - | - | 5.02±0.96 | 5.03±0.97 | 2.82±0.92 | 2.86±0.64 | - | - | - | - | 82±7 | 82±7 | 132±12 | 131.5±11.98 | - | - |
| Koc G [S21] | 2023 | 33(20-54) | Brown race | M/F | Patients with obesity and controls | 106 | 79 | - | - | 2.97±1.72 | 2.61± 1.5 | - | - | 2.50±0.35 | 2.51±0.35 | - | - | - | - | - | - | - | - | - | - |
| Zhang X [S22] | 2011 | 57.40±10.60 | Asian | M/F | Patients with T2DM | 49 | 463 | - | - | 2.18±0.62 | 2.43±2.00 | 4.98±1.21 | 4.92±1.34 | 3.61±1.33 | 3.85±1.56 | 1.05±0.58 | 1.03±0.58 | - | - | - | - | 91.8 ± 14.1 | 92±13.21 | - | - |
| Tretjakovs P [S23] | 2007 | 53.00±6.00 | Caucasian | M | Patients with CAD | 10 | 10 | 33.2±3.5 | 32.7± 2.0 | 1.01±0.13 | 1.18±0.29 | 4.87±1.25 | 5.83±1.40 | 3.62±1.39 | 3.32±1.01 | 1.17±0.24 | 1.31±0.17 | 5.22±0.5 | 5.28±0.61 | - | - | - | - | - | - |
| Tretjakovs P [S23] | 2007 | 53.00±7.00 | Caucasian | M | Healthy subjects | 10 | 10 | 23.7±1.1 | 25.9±1.9 | 1.94±0.96 | 2.05±0.88 | 4.97±0.84 | 5.33±1.16 | 3.92±1.06 | 3.95±0.99 | 1.36± 0.44 | 1.27±0.25 | 0.58±0.31 | 0.6±0.22 | - | - | - | - | - | - |
| Wernstedt I [S24] | 2004 | 56.80±0.31 | Caucasian | M/F | Patients with hypertension | 92 | 255 | - |  | 1.68 ±1.14 | 1.84±1.04 | 6.12±1.06 | 6.14±1.14 | 3.2±0.98 | 3.21±0.83 | 1.33±0.48 | 1.28±0.42 | 4.99±0.36 | 5.17±0.34 | - | - | 172±15.35 | 173.35±15.93 | - | - |
| Teixeira AA [S25] | 2015 | 57.30±10.10 | Brown race | M/F | Patients with hypertension | 373 | 239 | 28.9±5.1 | 30.1±5.4 | 1.8±0.8 | 1.77±0.79 | 2.39±0.49 | 2.39±0.42 | - | - | 1.52±0.4 | 1.43±0.4 | 9.11±3.34 | 10.5±3.41 | 84.2±11.3 | 85.8±11.3 | 135 ± 19.5 | 138±20.6 | 95.0±12.3 | 97.1±12.2 |
| Buraczynska M [S26] | 2016 | 47.30±14.00 | Caucasian | M/F | Patients with T2DM | 316 | 774 | - | - | 1.52±0.78 | 2.02±1.57 | 4.8±1.2 | 4.9±1.43 | - | - | 1.4±0.6 | 1.47±0.87 | 5.13±0.37 | 5.3±0.4 | - | - | - | - | - | - |
| Cardellini M [S27] | 2005 | 39.00±12.00 | Caucasian | M/F | Patients with obesity | 37 | 40 | 43.6±5.3 | 46.8±7.78 | 2.21±1.44 | 1.97±1.3 | 4.89±1.06 | 5.63±0.96 | 1.29±0.44 | 1.31±0.44 | 1.22±0.31 | 1.27±0.54 | 5.18±0.5 | 5.18±0.48 | - | - | - | - | - | - |
| Nelson JE [S28] | 2016 | 46.20±12.00 | American | M/F | Patients with NAFLD | 211 | 391 | 34.6±6.7 | 34.8±6.3 | - | - | 5.09±1.1 | 5.04±1.07 | - | - | 1.13±0.29 | 1.16±0.31 | - | - | - | - | - | - | - | - |
| Sie MP [S29] | 2008 | 72.00±7.00 | Caucasian | M/F | Healthy subjects | 1390 | 2459 | 27±4 | 27±8.02 | - | - | 5.8±1.0 | 5.8±0.98 | 1.15±0.34 | 1.2±0.44 | 1.4 ± 0.4 | 1.4 ± 0.4 | - | - | 75±11 | 75±11 | 143 ± 21 | 143.26±21.75 | - | - |
| Papaoikonomou S [S30] | 2014 | 66.50±9.96 | Caucasian | M/F | Patients with T2DM | 113 | 95 | 28.49±4.869 | 29.1±4.652 | - | - | 4.59±0.94 | 4.79±1.2 | 1.28±0.4 | 1.33±0.47 | 1.23±0.34 | 1.19±0.34 | - | - | - | - | - | - | - | - |
| Papaoikonomou S [S30] | 2014 | 66.58±9.05 | Caucasian | M/F | Patients with T2DM | 89 | 114 | - | - | 1.76±0.82 | 2.01±0.93 | 5.03±1.03 | 5.05±1.14 | 2.98±1.18 | 3.03±1.01 | 1.33±0.34 | 1.28±0.35 | - | - | - | - | - | - |  |  |
| Bhatt SP [S31] | 2018 | 68.77±9.79 | Brown race | M/F | Patients with obesity | 113 | 58 | 36.5±13.0 | 38.98±12.84 | 1.78±0.45 | 1.9±1.09 | 4.47±1.14 | 4.92±1 | 2.54±0.94 | 2.73±0.94 | 1.07±0.2 | 1.06±0.21 | - | - | 85.9±8.1 | 84.74±14.05 | 132.5±12.3 | 133.49±11.89 | 104.9±15.5 | 106.95±12.53 |
| Bhatt SP [S31] | 2018 | 65.38±11.13 | Brown race | M/F | Patients with obesity | 44 | 25 | 30.1±6.7 | 33.22±7.23 | - | - | 4.43±0.73 | 5.01±1.16 | 4.14±1.18 | 4.1±1.18 | 1.18±0.27 | 1.07±0.16 | 5.53±1.82 | 5.47±2.25 | 129.7±9.3 | 131.42±18.22 | 129.7±9.3 | 131.42±18.22 | 100.8±10.4 | 106.2±12.12 |
| Lieb W [S32] | 2004 | 58.00±9.00 | Caucasian | M/F | Patients with CAD | 240 | 503 | 27.4±3.56 | 27.36±3.59 | - | - | 5.51±1.17 | 5.96±1.17 | 3.65±1.07 | 3.69±1.05 | 1.29±0.34 | 1.28±0.34 | 5.65±2.46 | 5.83±2.34 | 84±10.84 | 83.72±10.97 | 140±19.98 | 140±20.09 | - | - |
| Lieb W [S32] | 2004 | 56.00±7.00 | Caucasian | M/F | Patients with CAD | 211 | 368 | 28.5±3.78 | 28.41±3.74 | - | - | 6.13±1.22 | 6.25±1.22 | 3.7±1.1 | 3.71±1.1 | 1.27±0.38 | 1.32±0.38 | - | - | 84 ±10.46 | 84.28±10.42 | 133±16.85 | 132±16.77 | - | - |
| Lieb W [S32] | 2004 | 53.00±13.00 | Caucasian | M/F | Healthy subjects | 331 | 692 | 26.7±3.82 | 26.67±3.82 | 1.89±0.18 | 1.72±0.17 | 6.03±1.07 | 6.04±1.07 | 3.22±0.74 | 3.46±0.74 | 1.4±0.4 | 1.41±0.4 | - | - | 82 ±11.46 | 81.44±13.43 | 135±18.38 | 134 ±18.31 | - | - |
| Tütün U [S33] | 2006 | 29.30±4.30 | Other ethnicities | M/F | Patients with PCAD | 46 | 25 | - | - | - | - | - | - | - | - | - | - | 5.81±1.84 | 5.87±1.77 | - | - | - | - | - | - |
| Ansari WM [S34] | 2017 | 42.00±3.80 | Other ethnicities | M/F | Patients with PCAD | 478 | 172 | - | - | - | - | - | - | - | - | - | - | - | - | - | - | - | - | - | - |
| Maitra A [S35] | 2008 | 57.17±9.22 | Other ethnicities | M/F | Patients with PCAD | 3 | 83 | - | - | - | - | - | - | - | - | - | - | 8.52±4.11 | 8.57±4.01 | - | - | - | - | - | - |
| Sekuri C [S36] | 2007 | 46.30±7.80 | Other ethnicities | M/F | Patients with PCAD | 118 | 102 | - | - | - | - | - | - | - | - | - | - | - | - | - | - | - | - | - | - |
| Omer W [S37] | 2016 | 42.00±3.80 | Other ethnicities | M/F | Patients with PCAD | 478 | 172 | - | - | - | - | - | - | - | - | - | - | 5.56±0.88 | 6.08±2.34 | - | - | - | - | - | - |
| Galimudi RK [S38] | 2014 | 64.00±6.00 | Other ethnicities | M | Patients with PCAD | 185 | 215 | - | - | - | - | - | - | - | - | - | - | 5.39±1.4 | 5.42±1.15 | - | - | - | - | - | - |
| Jerrard-Dunne P [S39] | 2003 | 50-65 | Caucasian | M/F | Patients with PCAD | 341 | 763 | - | - | - | - | - | - | - | - | - | - | - | - | - | - | - | - | - | - |
| Coker A [S40] | 2011 | 53.40±9.50 | Other ethnicities | M/F | Patients with PCAD | 191 | 116 | - | - | - | - | - | - | - | - | - | - | - | - | - | - | - | - | - | - |

M: male; F: female; PCAD: premature coronary artery disease; CAD: coronary artery disease; T1DM: type 1 diabetes mellitus; T2DM: type 2 diabetes mellitus; CVD: cardiovascular disease; PCOS: polycystic ovary syndrome; NAFLD: nonalcoholic fatty liver disease; TG: triglycerides; TC: total cholesterol; LDL-C: low-density lipoprotein cholesterol; HDL-C: high-density lipoprotein cholesterol; FPG: fasting plasma glucose; SBP: systolic blood pressure; DBP: diastolic blood pressure; BMI: body mass index; WC: waist circumference.

**
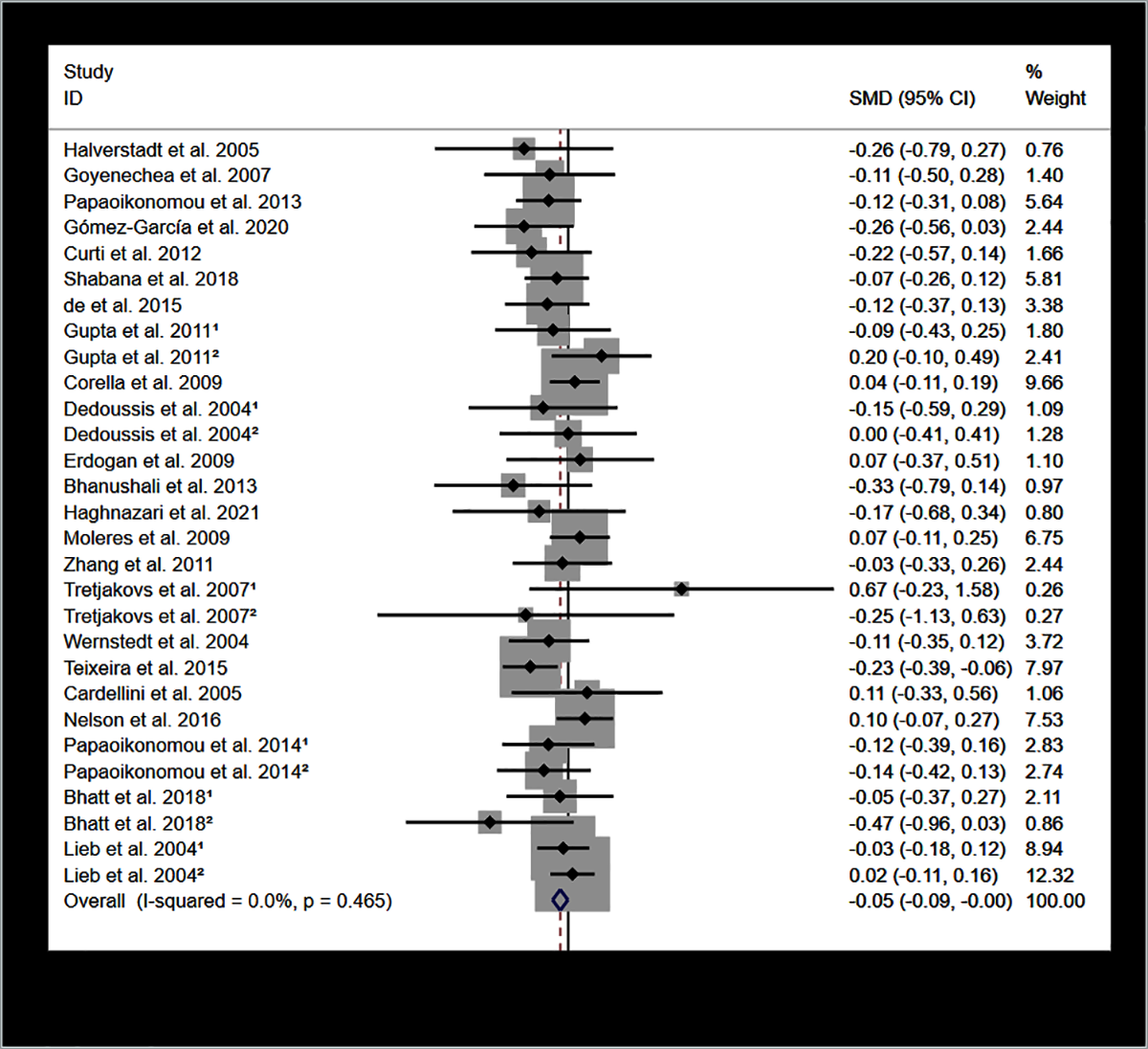
**

*Figure S1* **Forest plot of the meta-analysis between rs1800795 and high-density lipoprotein cholesterol levels.**


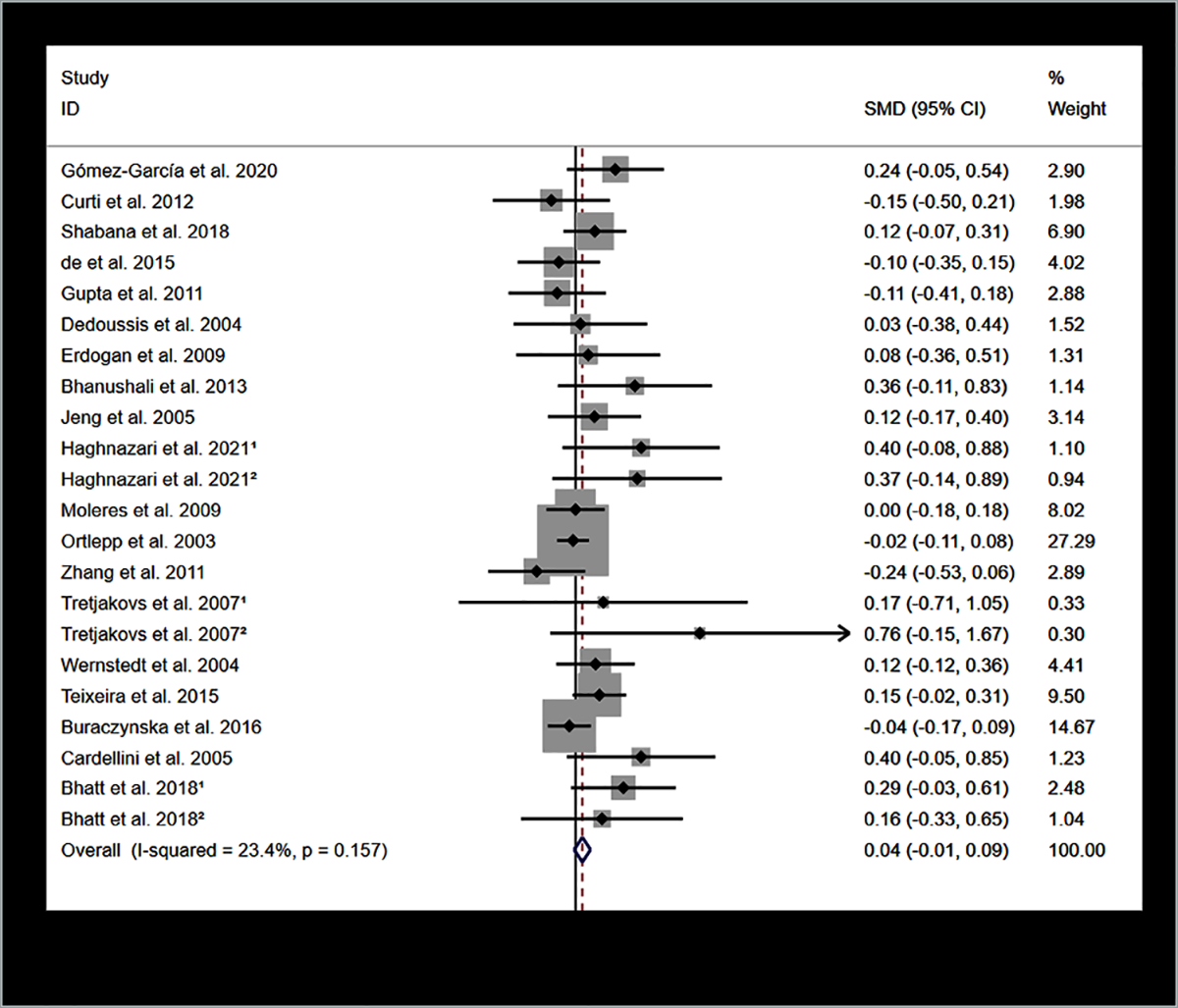


*Figure S2* **Forest plot of the meta-analysis between rs1800795 and triglycerides levels.**

*
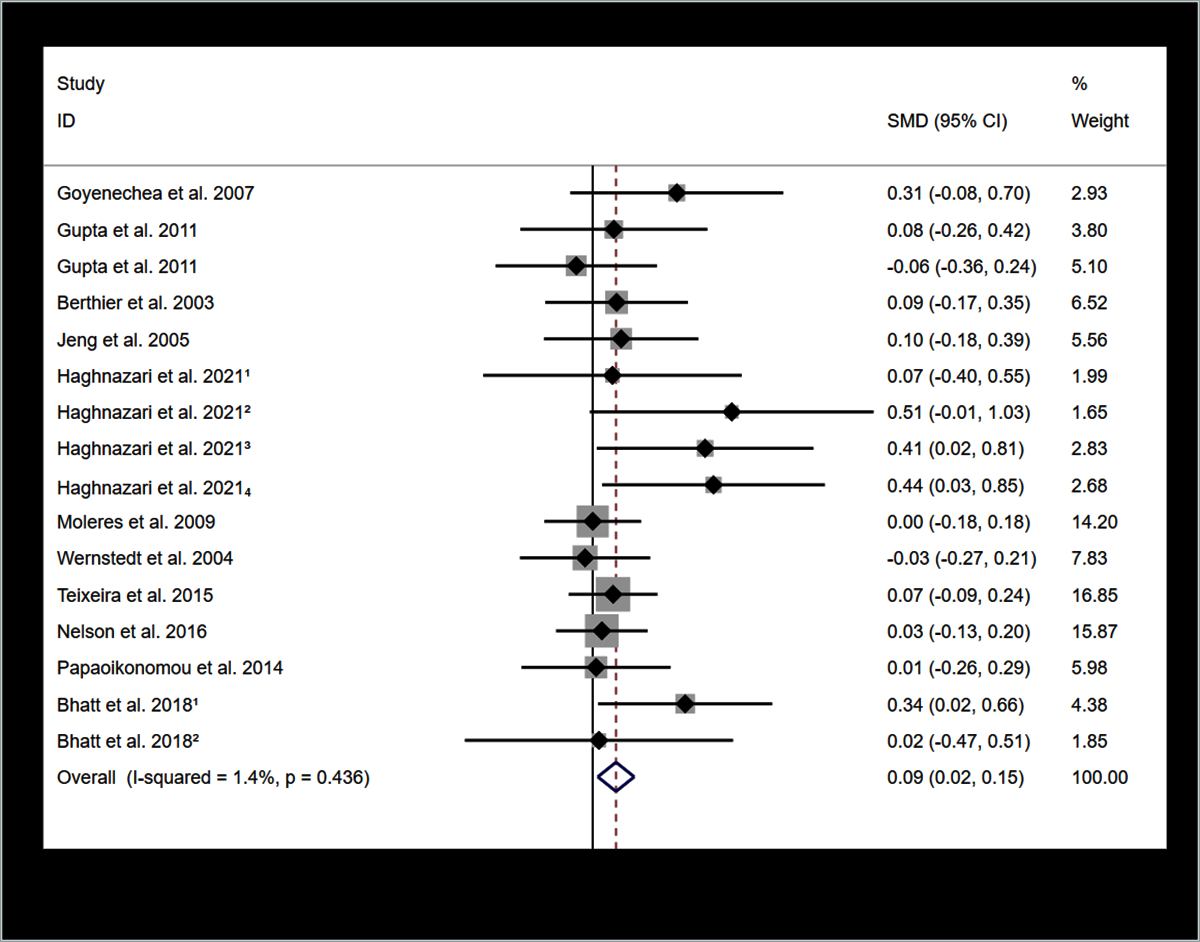
*

*Figure S3* **Forest plot of the meta-analysis between rs1800795 and fasting plasma glucose levels.**

**
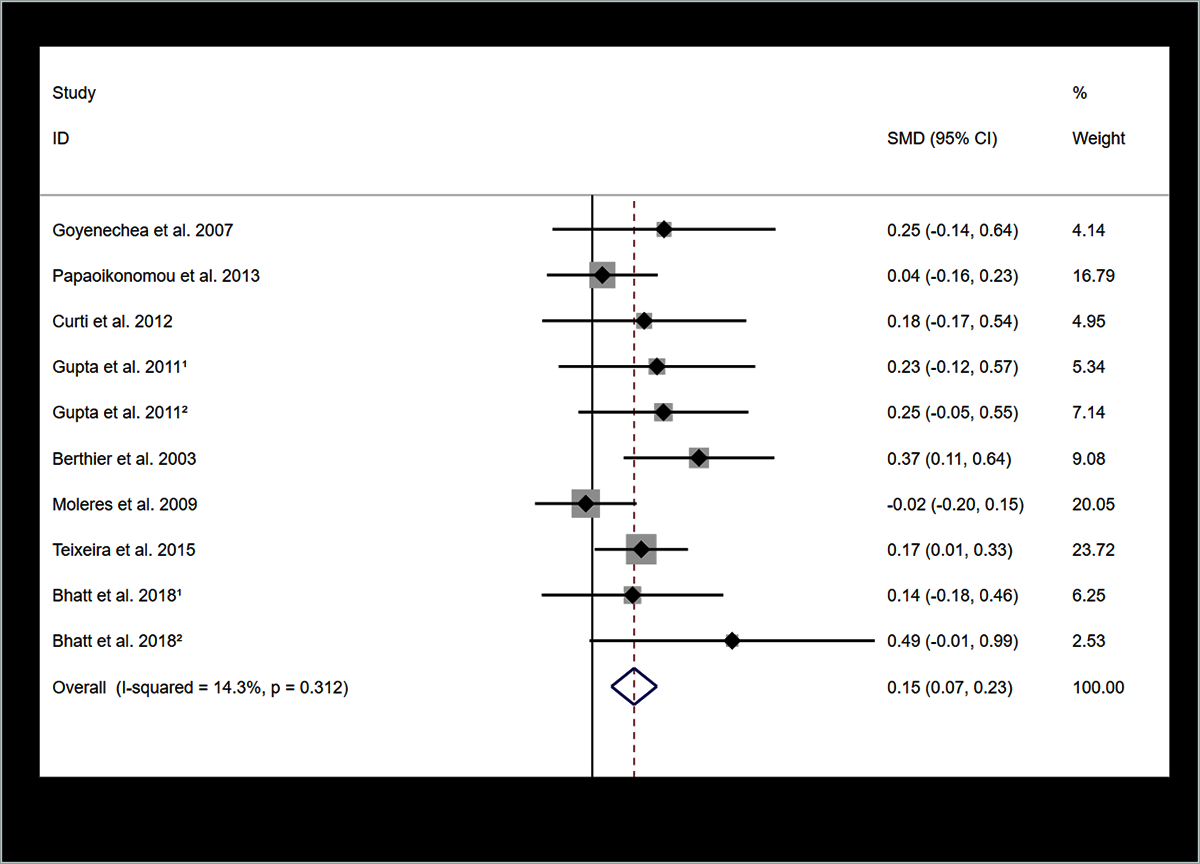
***Figure S4* **Forest plot of the meta-analysis between rs1800795 and waist circumference.**

**
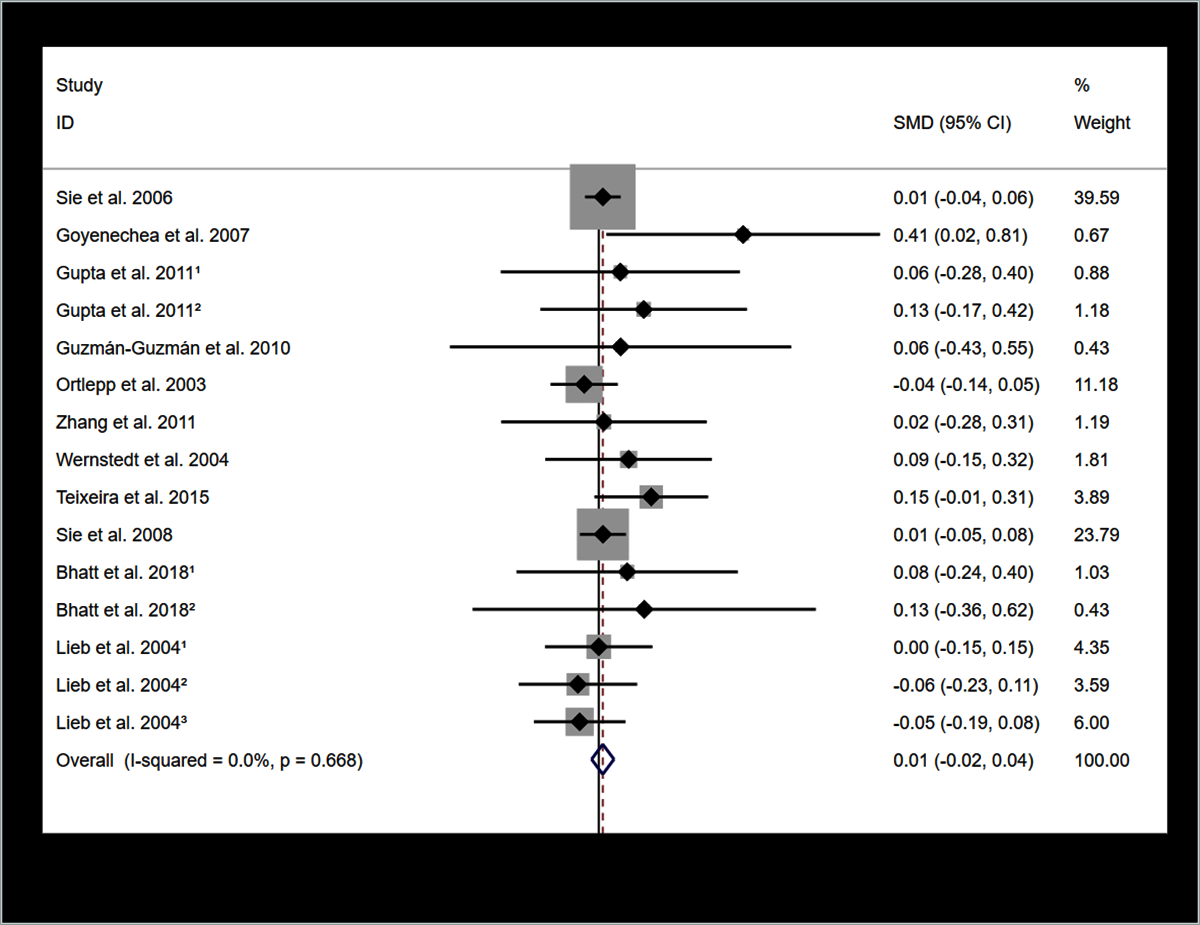
**

*Figure S5* **Forest plot of the meta-analysis between rs1800795 and systolic blood pressure.**

**
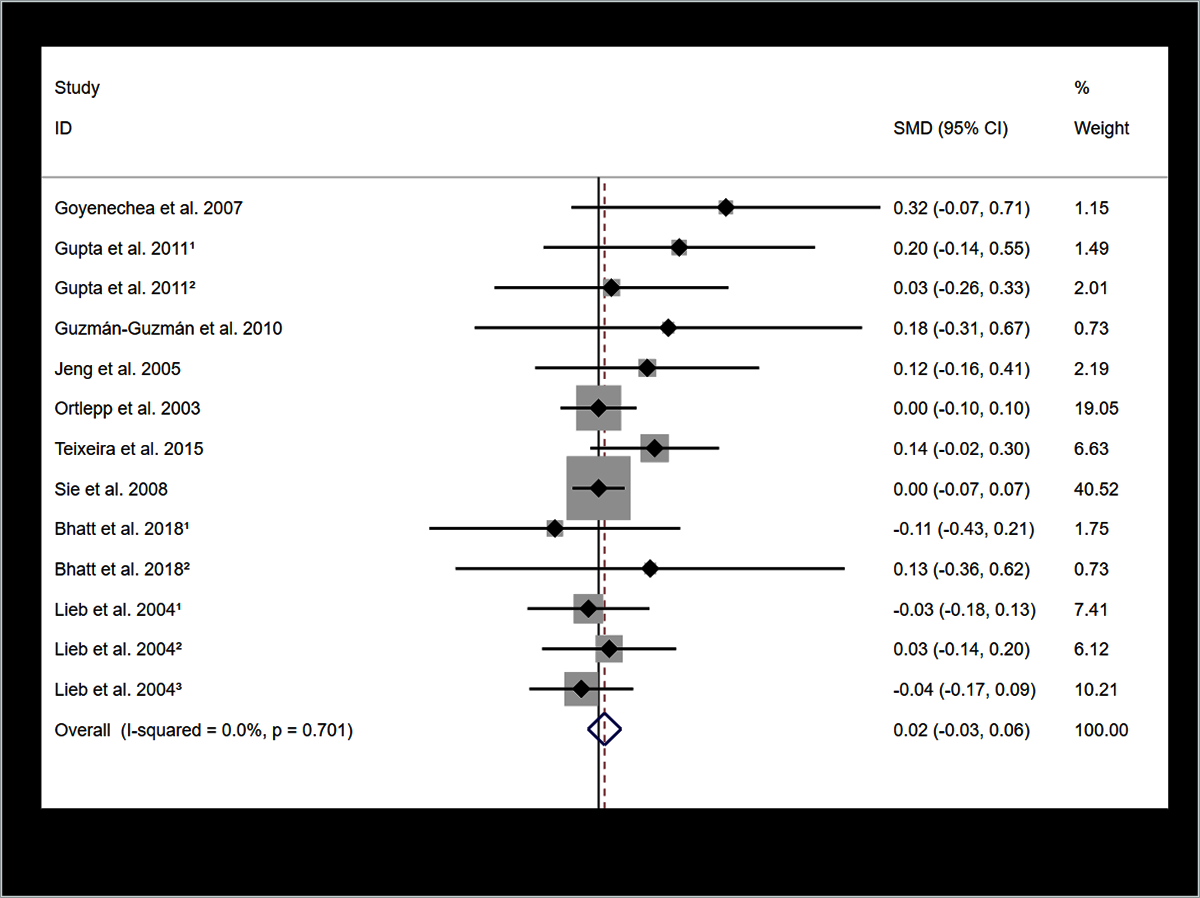
***Figure S6* **Forest plot of the meta-analysis between rs1800795 and diastolic blood pressure.**

**
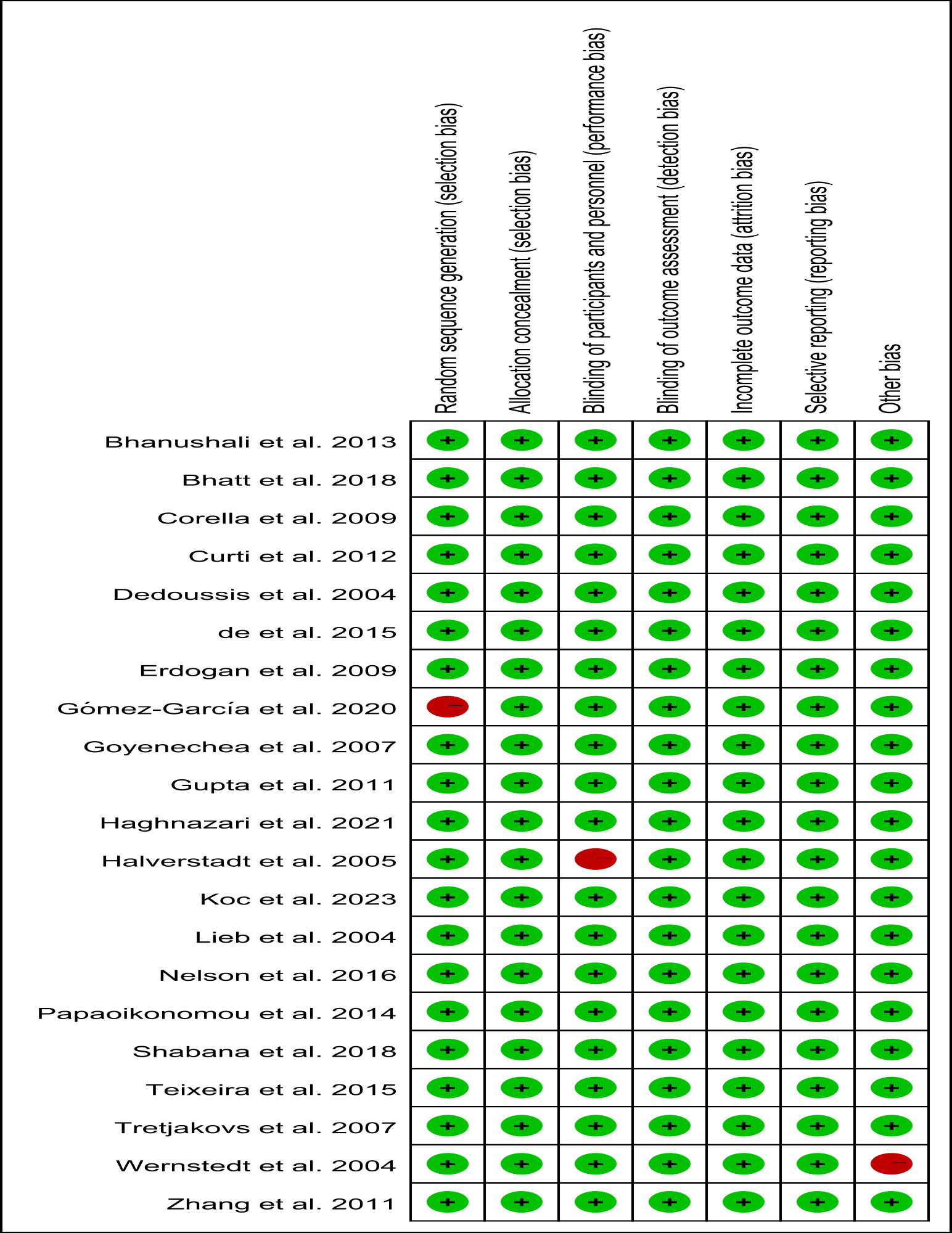
**

*Figure S7* **Risk bias plot of the meta-analysis between rs1800795 and low-density lipoprotein cholesterol levels**

**(for assessment of each entry, green represents low risk of bias and red refers to high risk of bias).**

**
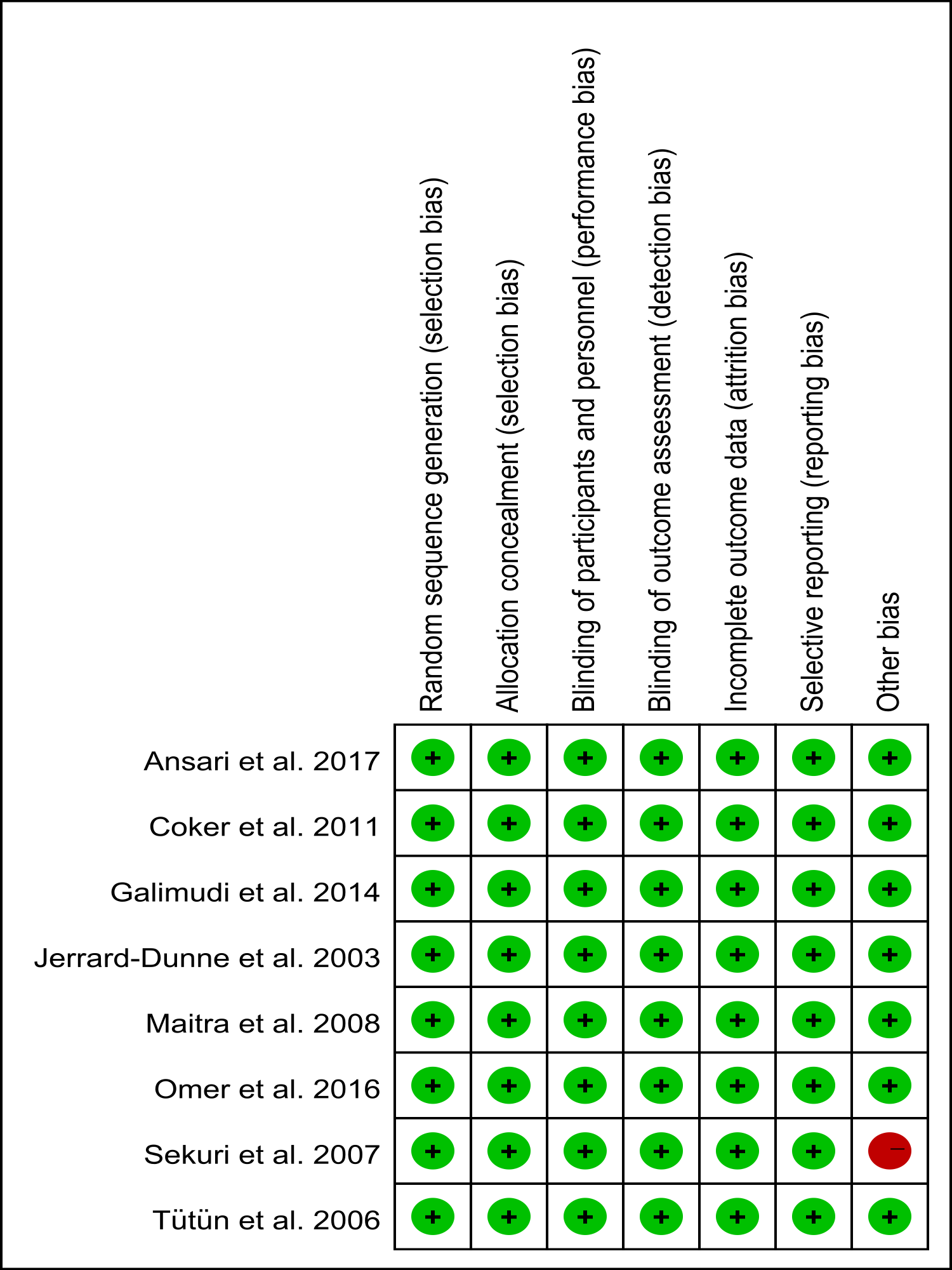
**

*Figure S8* **Risk bias plot of the meta-analysis between rs1800795 and premature coronary artery disease**

**(for assessment of each entry, green represents low risk of bias and red refers to high risk of bias).**

**References**

S1. Sie MP, Sayed-Tabatabaei FA, Oei HH, Uitterlinden AG, Pols HA, Hofman A, van Duijn CM, Witteman JC. Interleukin 6 -174 g/c promoter polymorphism and risk of coronary heart disease: results from the rotterdam study and a meta-analysis. Arterioscler Thromb Vasc Biol. 2006;26:212-7. doi: 10.1161/01.ATV.0000194099.65024.17.

S2. Halverstadt A, Phares DA, Roth S, Ferrell RE, Goldberg AP, Hagberg JM. Interleukin-6 genotype is associated with high-density lipoprotein cholesterol responses to exercise training. Biochim Biophys Acta. 2005;1734:143-51. doi: 10.1016/j.bbalip.2005.03.003.

S3. Goyenechea E, Parra D, Martínez JA. Impact of interleukin 6 -174G>C polymorphism on obesity-related metabolic disorders in people with excess in body weight. Metabolism. 2007;56(12):1643-8. doi: 10.1016/j.metabol.2007.07.005.

S4. Papaoikonomou S, Tentolouris N, Tousoulis D, Papadodiannis D, Miliou A, Papageorgiou N, Hatzis G, Stefanadis C. The association of the 174G>C polymorphism of interleukin 6 gene with diabetic nephropathy in patients with type 2 diabetes mellitus. J Diabetes Complications. 2013;27:576-9. doi: 10.1016/j.jdiacomp.2013.06.006.

S5. Gómez-García EF, Cortés-Sanabria L, Cueto-Manzano AM, Medina-Zavala RS, Hernández-Ramos LE, Martínez-Ramírez HR, Vásquez-Jiménez JC, Mendoza-Carrera F. Interactions Between Diet Quality and Interleukin-6 Genotypes Are Associated With Metabolic and Renal Function Parameters in Mexican Patients With Type 2 Diabetes Mellitus. J Ren Nutr. 2020;30:223-231. doi: 10.1053/j.jrn.2019.08.002.

S6. Curti ML, Pires MM, Barros CR, Siqueira-Catania A, Rogero MM, Ferreira SR. Associations of the TNF-alpha -308 G/A, IL6 -174 G/C and AdipoQ 45 T/G polymorphisms with inflammatory and metabolic responses to lifestyle intervention in Brazilians at high cardiometabolic risk. Diabetol Metab Syndr. 2012;4:49. doi: 10.1186/1758-5996-4-49.

S7. Shabana NA, Ashiq S, Ijaz A, Khalid F, Saadat IU, Khan K, Sarwar S, Shahid SU. Genetic risk score (GRS) constructed from polymorphisms in the PON1, IL-6, ITGB3, and ALDH2 genes is associated with the risk of coronary artery disease in Pakistani subjects. Lipids Health Dis. 2018;17:224. doi: 10.1186/s12944-018-0874-6.

S8. de Oliveira R, Moraes TI, Cerda A, Hirata MH, Fajardo CM, Sousa MC, Dorea EL, Bernik MM, Hirata RD. ADIPOQ and IL6 variants are associated with a pro-inflammatory status in obeses with cardiometabolic dysfunction. Diabetol Metab Syndr. 2015;7:34. doi: 10.1186/s13098-015-0027-2.

S9. Gupta A, Gupta V, Singh AK, Tiwari S, Agrawal S, Natu SM, Agrawal CG, Negi MP, Pant AB. Interleukin-6 G-174C gene polymorphism and serum resistin levels in North Indian women: potential risk of metabolic syndrome. Hum Exp Toxicol. 2011;30:1445-53. doi: 10.1177/0960327110393763.

S10. Berthier MT, Paradis AM, Tchernof A, Bergeron J, Prud'homme D, Després JP, Vohl MC. The interleukin 6-174G/C polymorphism is associated with indices of obesity in men. J Hum Genet. 2003;48:14-9. doi: 10.1007/s100380300002.

S11. Corella D, González JI, Bulló M, Carrasco P, Portolés O, Díez-Espino J, Covas MI, Ruíz-Gutierrez V, Gómez-Gracia E, Arós F, Fiol M, Herrera MC, Santos JM, Sáez G, Lamuela R, Lahoz C, Vinyoles E, Ros E, Estruch R. Polymorphisms cyclooxygenase-2 -765G>C and interleukin-6 -174G>C are associated with serum inflammation markers in a high cardiovascular risk population and do not modify the response to a Mediterranean diet supplemented with virgin olive oil or nuts. J Nutr. 2009;139:128-34. doi: 10.3945/jn.108.093054.

S12. Pramudji H, Demes CM, Dewi K, Tasmini T, Ahmad HS. Association of -174 G>C interleukin-6 gene polymorphism with interleukin-6 and c-reactive protein levels and obesity: A case-control study among people/residents of Western Indonesia. Med J Malaysia. 2019;74:400-404.

S13. Dedoussis GV, Manios Y, Choumerianou DM, Yiannakouris N, Panagiotakos DB, Skenderi K, Zampelas A. The IL-6 gene G-174C polymorphism related to health indices in Greek primary school children. Obes Res. 2004;12:1037-41. doi: 10.1038/oby.2004.130.

S14. Guzmán-Guzmán IP, Muñoz-Valle JF, Flores-Alfaro E, Salgado-Goytia L, Salgado-Bernabé AB, Parra-Rojas I. Interleukin-6 gene promoter polymorphisms and cardiovascular risk factors. A family study. Dis Markers. 2010;28:29-36. doi: 10.3233/DMA-2010-0680.

S15. Erdogan M, Karadeniz M, Berdeli A, Tamsel S, Yilmaz C. The relationship of the interleukin-6 -174 G>C gene polymorphism with cardiovascular risk factors in Turkish polycystic ovary syndrome patients. Int J Immunogenet. 2009;36:283-8. doi: 10.1111/j.1744-313X.2009.00867.x.

S16. Bhanushali AA, Das BR. Promoter variants in interleukin-6 and tumor necrosis factor alpha and risk of coronary artery disease in a population from Western India. Indian J Hum Genet. 2013;19:430-6. doi: 10.4103/0971-6866.124371.

S17. Jeng JR, Wang JH, Liu WS, Chen SP, Chen MY, Wu MH, Hsu WL, Lin SZ. Association of interleukin-6 gene G-174C polymorphism and plasma plasminogen activator inhibitor-1 level in Chinese patients with and without hypertension. Am J Hypertens. 2005;18):517-22. doi: 10.1016/j.amjhyper.2004.10.028.

S18. Haghnazari L, Sabzi R. Relationship between TP53 and interleukin-6 gene variants and the risk of types 1 and 2 diabetes mellitus development in the Kermanshah province. J Med Life. 2021;14(1):37-44. doi: 10.25122/jml-2019-0150.

S19. Moleres A, Rendo-Urteaga T, Azcona C, Martínez JA, Gómez-Martínez S, Ruiz JR, Moreno LA, Marcos A, Marti A; AVENA group. Il6 gene promoter polymorphism (-174G/C) influences the association between fat mass and cardiovascular risk factors. J Physiol Biochem. 2009;65(4):405-13. doi: 10.1007/BF03185936.

S20. Ortlepp JR, Metrikat J, Vesper K, Mevissen V, Schmitz F, Albrecht M, Maya-Pelzer P, Hanrath P, Weber C, Zerres K, Hoffmann R. The interleukin-6 promoter polymorphism is associated with elevated leukocyte, lymphocyte, and monocyte counts and reduced physical fitness in young healthy smokers. J Mol Med (Berl). 2003;81:578-84. doi: 10.1007/s00109-003-0471-6. Epub 2003 Aug 20.

S21. Koc G, Doran T, Uygur MM, Kirac D. Obesity is associated with IL-6 gene polymorphisms rs1800795 and rs1800796 but not SOCS3 rs4969170. Mol Biol Rep. 2023;50:2041-2048. doi: 10.1007/s11033-022-08129-y.

S22. Zhang X, Ma L, Peng F, Wu Y, Chen Y, Yu L, Lei Z, Zhang C. The endothelial dysfunction in patients with type 2 diabetes mellitus is associated with IL-6 gene promoter polymorphism in Chinese population. Endocrine. 2011;40:124-9. doi: 10.1007/s12020-011-9442-9.

S23. Tretjakovs P, Latkovskis G, Licis N, Juhnevica D, Jurka A, Bormane I, Aivars JI, Stifts A, Pirags V. Interleukin-6 gene promoter -174G/C polymorphism and insulin resistance: a pilot study. Clin Chem Lab Med. 2007;45:1145-8. doi: 10.1515/CCLM.2007.260.

S24. Wernstedt I, Eriksson AL, Berndtsson A, Hoffstedt J, Skrtic S, Hedner T, Hultén LM, Wiklund O, Ohlsson C, Jansson JO. A common polymorphism in the interleukin-6 gene promoter is associated with overweight. Int J Obes Relat Metab Disord. 2004;28:1272-9. doi: 10.1038/sj.ijo.0802763.

S25. Teixeira AA, Quinto BM, Dalboni MA, Rodrigues CJ, Batista MC. Association of IL-6 polymorphism -174G/C and metabolic syndrome in hypertensive patients. Biomed Res Int. 2015;2015:927589. doi: 10.1155/2015/927589.

S26. Buraczynska M, Zukowski P, Drop B, Baranowicz-Gaszczyk I, Ksiazek A. Effect of G(-174)C polymorphism in interleukin-6 gene on cardiovascular disease in type 2 diabetes patients. Cytokine. 2016;79:7-11. doi: 10.1016/j.cyto.2015.12.004.

S27. Cardellini M, Perego L, D'Adamo M, Marini MA, Procopio C, Hribal ML, Andreozzi F, Frontoni S, Giacomelli M, Paganelli M, Pontiroli AE, Lauro R, Folli F, Sesti G. C-174G polymorphism in the promoter of the interleukin-6 gene is associated with insulin resistance. Diabetes Care. 2005;28:2007-12. doi: 10.2337/diacare.28.8.2007.

S28. Nelson JE, Handa P, Aouizerat B, Wilson L, Vemulakonda LA, Yeh MM, Kowdley KV; NASH Clinical Research Network. Increased parenchymal damage and steatohepatitis in Caucasian non-alcoholic fatty liver disease patients with common IL1B and IL6 polymorphisms. Aliment Pharmacol Ther. 2016;44:1253-1264. doi: 10.1111/apt.13824.

S29. Sie MP, Mattace-Raso FU, Uitterlinden AG, Arp PP, Hofman A, Pols HA, Hoeks AP, Reneman RS, Asmar R, van Duijn CM, Witteman JC. The interleukin-6-174 G/C promoter polymorphism and arterial stiffness; the Rotterdam Study. Vasc Health Risk Manag. 2008;4:863-9. doi: 10.2147/vhrm.s1693.

S30. Papaoikonomou S, Tousoulis D, Tentolouris N, Miliou A, Papageorgiou N, Synetos A, Stefanadis C. Assessment of the effects of 174G/C polymorphism on interleukin 6 gene on macrovascular complications in patients with type 2 diabetes mellitus. Int J Cardiol. 2014;172:e190-1. doi: 10.1016/j.ijcard.2013.12.135.

S31. Bhatt SP, Guleria R, Vikram NK, Vivekanandhan S, Singh Y, Gupta AK. Association of inflammatory genes in obstructive sleep apnea and non alcoholic fatty liver disease in Asian Indians residing in north India. PLoS One. 2018;13:e0199599. doi: 10.1371/journal.pone.0199599. eCollection 2018.

S32. Lieb W, Pavlik R, Erdmann J, Mayer B, Holmer SR, Fischer M, Baessler A, Hengstenberg C, Loewel H, Doering A, Riegger GA, Schunkert H. No association of interleukin-6 gene polymorphism (-174 G/C) with myocardial infarction or traditional cardiovascular risk factors. Int J Cardiol. 2004;97(2):205-12. doi: 10.1016/j.ijcard.2003.07.038.

S33. Tütün U, Aksöyek A, Ulus AT, Misirlioğlu M, Ciçekçioğlu F, Ozişik K, Ihsan Parlar A, Baran Budak A, Gedik S, Katircioğlu SF. Gene polymorphisms in patients below 35 years of age who underwent coronary artery bypass surgery. Coron Artery Dis. 2006;17(1):35-9. doi: 10.1097/00019501-200602000-00006.

S34. Ansari WM, Humphries SE, Naveed AK, Khan OJ, Khan DA. Influence of cytokine gene polymorphisms on proinflammatory/anti-inflammatory cytokine imbalance in premature coronary artery disease. Postgrad Med J. 2017;93:209-214. doi: 10.1136/postgradmedj-2016-134167.

S35. Maitra A, Shanker J, Dash D, John S, Sannappa PR, Rao VS, Ramanna JK, Kakkar VV. Polymorphisms in the IL6 gene in Asian Indian families with premature coronary artery disease--the Indian Atherosclerosis Research Study. Thromb Haemost. 2008;99:944-50. doi: 10.1160/TH07-11-0686.

S36. Sekuri C, Cam FS, Sagcan A, Ercan E, Tengiz I, Alioglu E, Berdeli A. No association of interleukin-6 gene polymorphism (-174 G/C) with premature coronary artery disease in a Turkish cohort. Coron Artery Dis. 2007;18:333-7. doi: 10.1097/MCA.0b013e32820588ae.

S37. Omer W, Naveed AK, Khan OJ, Khan DA. Role of Cytokine Gene Score in Risk Prediction of Premature Coronary Artery Disease. Genet Test Mol Biomarkers. 2016;20:685-691. doi: 10.1089/gtmb.2016.0108.

S38. Galimudi RK, Spurthi MK, Padala C, Kumar KG, Mudigonda S, Reddy SG, Aiyengar MT, Sahu SK, Rani SH. Interleukin 6(-174G/C) variant and its circulating levels in coronary artery disease patients and their first degree relatives. Inflammation. 2014;37(2):314-21. doi: 10.1007/s10753-013-9742-8.

S39. Jerrard-Dunne P, Sitzer M, Risley P, Steckel DA, Buehler A, von Kegler S, Markus HS; Carotid Atherosclerosis Progression Study. Interleukin-6 promoter polymorphism modulates the effects of heavy alcohol consumption on early carotid artery atherosclerosis: the Carotid Atherosclerosis Progression Study (CAPS). Stroke. 2003;34:402-7. doi: 10.1161/01.str.0000053849.09308.b2.

S40. Coker A, Arman A, Soylu O, Tezel T, Yildirim A. Lack of association between IL-1 and IL-6 gene polymorphisms and myocardial infarction in Turkish population. Int J Immunogenet. 2011;38:201-8. doi: 10.1111/j.1744-313X.2010.00988.x.
